# Effects of whole grains on glycemic control: a systematic review and dose-response meta-analysis of prospective cohort studies and randomized controlled trials

**DOI:** 10.1186/s12937-024-00952-2

**Published:** 2024-04-25

**Authors:** Tao Ying, Jianheng Zheng, Juntao Kan, Wenyun Li, Kun Xue, Jun Du, Yuwei Liu, Gengsheng He

**Affiliations:** 1 School of Public Health, Key Laboratory of Public Health Safety of the Ministry of Education, Fudan University, 130 Dong’an Road, Shanghai, 200032 China; 2 Nutrilite Health Institute, Shanghai, China

**Keywords:** Dose-response, Glycemic control, Meta-analysis, Recommendations, Type 2 diabetes, Whole grains

## Abstract

**Purpose:**

Whole grains have recently been promoted as beneficial to diabetes prevention. However, the evidence for the glycemic benefits of whole grains seems to conflict between the cohort studies and randomized control trials (RCTs). To fill the research gap, we conducted a meta-analysis to determine the effects of whole grains on diabetes prevention and to inform recommendations.

**Methods:**

We searched PubMed, Clarivate Web of Science, and Cochrane Library until March 2024. We used the risk ratio (RR) of type 2 diabetes to represent the clinical outcomes for cohort studies, while the biomarkers, including fasting blood glucose and insulin, HbA_1C_, and HOMA-IR, were utilized to show outcomes for RCTs. Dose-response relationships between whole grain intakes and outcomes were tested with random effects meta-regression models and restricted cubic splines models. This study is registered with PROSPERO, CRD42021281639.

**Results:**

Ten prospective cohort studies and 37 RCTs were included. Cohort studies suggested a 50 g/day whole grain intake reduced the risk of type 2 diabetes (RR = 0.761, 95% CI: 0.700 to 0.828, *I*^*2*^ = 72.39%, *P* < 0.001) and indicated a monotonic inverse relationship between whole grains and type 2 diabetes rate. In RCTs, whole grains significantly reduced fasting blood glucose (Mean difference (MD) = -0.103 mmol/L, 95% CI: -0.178 to -0.028; *I*^*2*^ = 72.99%, *P* < 0.01) and had modest effects on HbA_1C_ (MD = -0.662 mmol/mol (-0.06%), 95% CI: -1.335 to 0.010; *I*^*2*^ = 64.55%, *P* = 0.05) and HOMA-IR (MD = -0.164, 95% CI: -0.342 to 0.013; *I*^*2*^ = 33.38%, *P* = 0.07). The intake of whole grains and FBG, HbA_1C_, and HOMA-IR were significantly dose-dependent. The restricted spline curves remained flat up to 150 g/day and decreased afterward. Subgroup analysis showed that interventions with multiple whole-grain types were more effective than those with a single type.

**Conclusion:**

Our study findings suggest that a daily intake of more than 150 g of whole grain ingredients is recommended as a population approach for diabetes prevention.

**Supplementary Information:**

The online version contains supplementary material available at 10.1186/s12937-024-00952-2.

## Introduction

Type 2 diabetes is a major global concern for human health and life expenditure. According to the International Diabetes Federation, an estimated 536.6 million people have been living with the rising burden of diabetes since 2021 [1]. Type 2 diabetes has several causes, among which unhealthy diets have been recognized as one of the most paramount contributors to the current global epidemic. In particular, individuals consuming carbohydrates of poor quality are linked to a higher risk of developing type 2 diabetes with the estimation that grains contribute approximately half of daily calorie intakes [2]. Thus, whole grains, often considered higher-quality sources of carbohydrates, have been highly promoted for their glycemic benefits [2, 3]. Defined by the HEALTHGRAIN Consortium, whole grains are intact, ground, cracked, or flaked grain kernels that contain all three anatomical components (endosperm, bran, and germ) in their original proportions [4]. To date, dietary strategies that focus on grain intake are still limited, especially for whole grains. While the current guidelines are only available to a general population and are irrespective of the risk level of individual health outcome [5–10], specific recommendations targeting type 2 diabetes prevention and management are urgently needed.

Shaping the population-level whole grain guidelines into a preventive recommendation for type 2 diabetes is a challenging goal that demands building an evidence base for observational studies and interventions. Previous reviews have evaluated evidence from prospective cohort studies and RCTs, proposing that the strength of the evidence from observational studies should support promoting whole grains for type 2 diabetes prevention [11–14]. However, the intervention effects in RCTs remain conflicting and not as pronounced as in cohort studies [15–19]. The variations in the doses and types of whole grains (mixed vs. single) and the health status of participants (healthy vs. metabolically abnormal) contribute to considerable variations in glycemic impacts. For example, consuming different types of whole grains might result in diverse metabolic outcomes because β-glucan in oats may barely slow the absorption of carbohydrates. At the same time, arabinoxylan rich in rye and wheat may increase the gut energy excretion [20]. Besides, the postulated dose-dependency of whole grains has rarely been investigated in RCTs to capture a sufficient dose on glycemic control, mostly due to ununified calculations of whole grain ingredients across products [13, 21]. Considering the contextual complexity of whole grain interventions, while numerous efforts have been made in intervention evaluations [22–26], the evidence to date would not be sufficient to reshape the dietary guidelines to reduce population type 2 diabetes risk. Thus, a combination of systematic and dose-dependent evaluations that elucidate the effectiveness and heterogeneity of RCTs would greatly help the continuing development of whole grain recommendations for type 2 diabetes.

Hence, this systematic review and meta-analysis aimed to evaluate the association between whole grain intake and incidence of type 2 diabetes in prospective cohort studies and then the effects of whole grains on the markers of glycemic control for RCTs. Subgroup analysis and dose-response curve would also be formulated to help illustrate the differences between cohort studies and RCTs and derive a quantitative recommendation for the daily consumption of whole grain ingredients to prevent type 2 diabetes.

## Materials and methods

### Protocol and data collection

We performed a systematic review and meta-analysis of prospective cohort studies that assessed the effects of whole grains on the occurrence of type 2 diabetes and RCTs that evaluated whole grain consumption on glycemic control. The protocol of the systematic review has been published on the PROSPERO register (http://www.crd.york.ac.uk/prospero/) under registration number CRD42021281639. PICO strategy (Supplementary Table 1) and detailed methods are presented in the electronic supplementary material (ESM) Methods. To summarize, we searched PubMed, Clarivate Web of Science, and Cochrane Library until March 2024. Eligible studies were those that examined the effect of whole grains on the risk of type 2 diabetes for prospective cohort studies or intermediate glycemic biomarkers for RCTs. The quality of the cohort studies was assessed using the well-established Newcastle-Ottawa Scale (NOS) tool [26], while the Cochrane Risk of Bias Assessment tool [27] was utilized to evaluate the quality of the RCTs (Supplementary Tables 2 and 3).

### Data synthesis

Random-effects models were utilized to calculate the overall effect size using risk ratios (RRs) and hazard ratios (HRs) for assessing the occurrence of type 2 diabetes and mean differences for glycemic biomarkers. *I*^*2*^ statistic was used to assess between-study heterogeneity; a value over 50% indicated a significant level of heterogeneity. One-study-removed sensitivity analyses were obtained to determine whether removing any study could cause significant changes to the results. The possibility of a publication bias was examined by the visual inspection of funnel plots and the application of Begg’s test. For the dose-response analysis, all whole grain intakes were transformed into whole grain ingredients in g/day and tested with random effects meta-regression models and restricted cubic splines models [13, 14].

For RCTs, we did further subgroup analysis to detect probable sources of heterogeneity with a random effects model. Subgroup analyses included stratification for lengths of intervention (< 12 weeks or ≥ 12 weeks), types of study design (parallel or crossover), whole grain products variety (1–2 types or ≥ 5 types of whole grain products), types of whole grain (wheat, rice, mix or others), health status (generally healthy or unhealthy), baseline BMI, baseline age, baseline triglycerides and quality of studies (low risk, uncertain and high risk). Types of whole grain products refer to several major food categories containing whole grain ingredients, including bread (bread rolls, muffins, biscuits, etc.), cereal (ready-to-eat and hot cereal), grains (pasta, rice, etc.), grain-based desserts (cookies, cakes, pies, chips, etc.) and mixed dishes (pizza, salads, etc.). The “Generally healthy subgroup” included healthy individuals as well as overweight or obese people, and the “unhealthy subgroup” had studies on individuals with pre-diabetes, diabetes, metabolic syndrome, or at risk of metabolic disease (participants with at least one impaired glucose, lipid, or blood pressure). All analyses were conducted using R 4.0.2 software with “metafor” and “dosresmeta” packages. P values < 0.05 were considered statistically significant.

## Results

### Flow and characteristics of the included studies

**Fig. 1:**
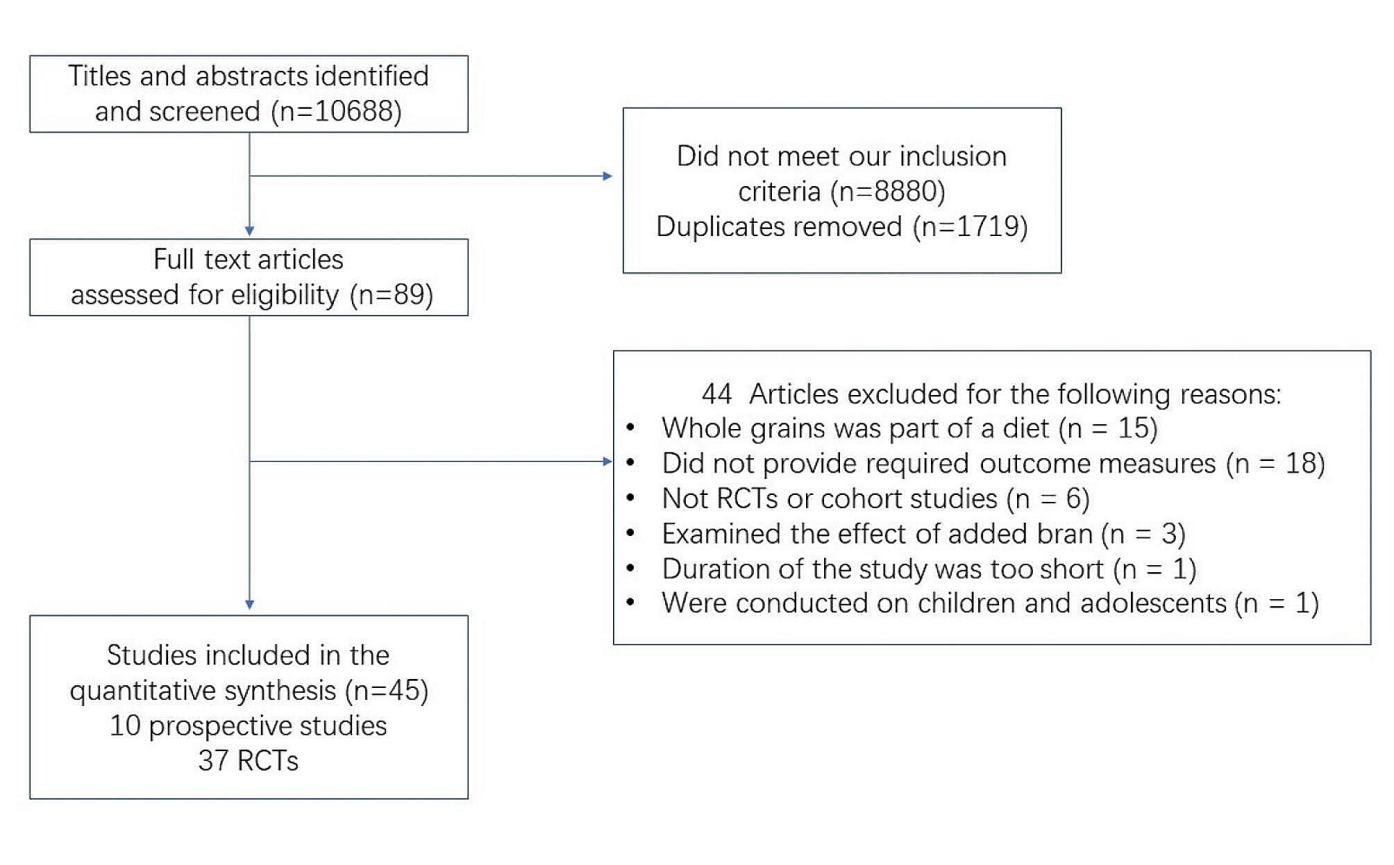
Flow chart indicating the process by which eligible prospective cohort studies and RCTs were identified

A flow chart of study identification was shown in Fig. 1. Ten prospective cohort studies with 473,019 adults [27–34] and 37 RCTs with 3136 participants [22–24, 35–68] were included in the meta-analyses. Characteristics of the included studies were shown in Tables 1 and 2. For cohort studies, 6 studies were performed in the United States [27, 29, 33, 34], 2 in the Sweden Field [30, 32], 1 in the Finland Field [28], and 1 in the Denmark [32]. Follow-up years ranged from 6 to 40 years, and whole grain ingredient consumption ranged from 0.15 g/day to 151 g/day. There were 7 cohort studies classified as low risk of bias and 3 with moderate risk of bias (Supplemental Table 1).

**Table 1:** Characteristics of prospective cohort studies included in the meta-analysis of whole grain intake and type 2 diabetes in adults

Author, year	Cohort	Follow time, follow-year, sex, country	Study size, age, cases	Dietary assessment method	Method of reporting whole grain intakes (unit)	Low whole grain intake, high whole grain intake	RR (95%CI) for highest dose group compared with lowest dose group	adjustments for confounders
Li et al., 2022 [34]	Women’s Health Initiative Observational Study	1993/1998–2020, 15.8, F, USA	108,681 w, mean age 63 years at baseline: 15,842 cases	validated FFQ, 122 food items	ingredient (g/day)	6.3 g/day, 78.5 g/day	0.84(0.79, 0.88)	Age, residence, energy intake, race/ethnicity, physical activity, smoking status, pack-years of cigarettes, alcohol, HRT, education, income, family history of diabetes, antihypertensive medication use, dietary protein sources, BMI
Hu et al., 2020a [33]	Nurses’ health study	1976–2014, 40, F, USA	69,139 w, 30–55 years: 8170 cases	validated FFQ, 116 items	ingredient (servings/day)	0.1 servings/day, 1.9 servings/day	0.68(0.63, 0.73)	Age, ethnicity, smoking, alcohol, multivitamin use, physical activity, modified alternative healthy eating index, family history of diabetes, HRT, oral contraceptive use, BMI
Hu et al., 2020b [33]	Nurses’ Health study II	1989–2017, 28, F, USA	89,120 w, 25–42 years: 7072 cases	validated FFQ, 116 items	ingredient (servings/day)	0.3 servings/day, 2.5 servings/day	0.73(0.68, 0.8)	Age, ethnicity, smoking, alcohol, multivitamin use, physical activity, modified alternative healthy eating index, family history of diabetes, HRT, oral contraceptive use, BMI
Hu et al., 2020c [33]	Health Professionals Follow-up study	1986–2016, 30, M, USA	36,525 m, 40–75 years: 3387 cases	validated FFQ, 116 items	ingredient (servings/day)	0.2 servings/day, 2.8 servings/day	0.72(0.64, 0.81)	Age, ethnicity, smoking, multivitamin use, physical activity, modified alternative healthy eating index, family history of diabetes, BMI
Kyro et al., 2018 [32]	Danish Diet, Cancer, and Health cohort	1993/1997–2018,15, M&F, Denmark	26,251 m & 29,214 w, 50–65 years: 7417	validated FFQ, 192 food items	ingredient (g/day)	M: 19.45 g/day, 68.85 g/day; F: 19.05 g/day, 59.1 g/day	M: 0.8(0.73, 0.88); F: 0.85(0.77, 0.94)	Age, education, physical activity, smoking, alcohol, red processed meat, menopause, HRT, BMI
Wirstrom et al., 2013 [31]	Swedish middle-aged men and women	1992/1998–2013, 8–10, M&F, Sweden	5477 m/w, 35–56 years: 165 cases	validated FFQ, NA	ingredient (g/day)	16.35 g/day, 73.35 g/day	0.71(0.48, 1.04)	Age, sex, parental history of diabetes, BMI, physical activity, smoking, education, blood pressure
Ericson et al., 2013 [30]	Malmo Diet and Cancer Cohort	1991/1996–2006, 12, M&F, Sweden	27,140 m/w, 45–74 years: 1709 cases	validated diet history, FFQ, 168 food items	products (servings/day)	M: 1 servings/day, 2 servings/day; F: 0.01 servings/day, 2.3 servings/day	M: 0.83(0.67, 1.03); F: 0.85(0.68, 1.06)	Age, dietary method, season, total energy, education, smoking, alcohol, physical activity, BMI
van Dam et al., 2006 [29]	Black Women’s Health Study	1995–2003, 8, F, USA	41,186 w, age 21–69 years: 1964 cases	validated FFQ, 68 food items	products (serving/day)	0.03 servings/day, 1.29 servings/day	0.69(0.6, 0.79)	Age, total energy intake, BMI, smoking, physical activity, alcohol, parental history of diabetes, education, coffee, sugar sweetened soft drink, processed meat, red meat, low-fat dairy
Montonen et al., 2003 [28]	Finnish Mobile Clinic Health Examination Survey	1966/1972–1995, 23, M&F, Finland	2268 m &2030 w, 40–69 years: 52/102 cases	baseline FFQ, > 100 food items	products (g/day)	79 g/day, 302 g/day	0.65(0.36, 1.18)	Age, sex, geographic area, smoking, BMI, total energy intake, fruit, berries and vegetables
Mayer et al., 2000 [27]	Iowa Women’s Health Study	1986–1992, 6, F, USA	35,988 w, 55–69 years: 1141 cases	validated FFQ, 127 food items	products (servings/week)	1 servings/week, 20.5 servings/week	0.79(0.65, 0.96)	Age, total energy intake, BMI, WHR, education, smoking, alcohol, physical activity

^a^BMI, body mass index; F&w, female; FFQ, food frequency questionnaire; HRT, hormone replacement therapy; M&m, male; WHR, waist-to-hip ratio

**Table 2:** Characteristics of RCTs included in the meta-analysis of whole grain intake and glycemic control in adults

Author, year	Design	Sample size	Sex	Country	Age^b^	Baseline BMI^b^	Health status	Duration (week)	Whole grains in intervention groups	Refined grains in control groups	Whole grain products variety	Measured biomarkers
Ding et al. 2022 [68]	parallel	I: 45; C: 54	F:40; M:55	China	I: 64.9 ± 4.09; C: 64.8 ± 6.47	I: 25.7 ± 2.9; C: 25.6 ± 3.5	T2DM	12	100 g/day germinated brown rice	100 g/day refined white rice	1–2	FBG, HbA_1C_
Xue et al., 2021 [67]	parallel	I: 80; C: 78	F: 126; M: 32	China	I: 44.5 ± 12.5; C: 45.4 ± 14.0	I: 24.3 ± 3.9; C: 24.1 ± 4.2	healthy/overweight	12	154 g/d whole grain rye crisp bread + whole grain rye puffs	142.8 g refined wheat crisp + refined wheat puffs	1–2	FBG, FBI, HOMA-IR, HbA_1C_
Ren et al., 2020 [66]	parallel	I1: 28; I2: 28; I3: 28; C: 28	NA	China	18–70	NA	MetS	4	I1: 50 g Paddy rice with the husk removed + 50 g polished rice I2: 50 g Hot-air drying germinated brown rice + 50 g polished rice I3: 50 g autoclaving germinated brown rice + 50 g polished rice	100 g polished rice	1–2	FBG
Mai et al., 2020 [65]	parallel	I: 40; C: 40	F: 64; M: 16	Vietnam	I: 65.2 ± 3.78; C: 65.0 ± 3.85	I: 25.9 ± 2.4; C: 25.5 ± 2.3	MetS	12	200 g cooked pregerminated brown rice	200 g white rice	1–2	FBG, FBI, HOMA-IR
Roager et al., 2019 [64]	crossover	50	F: 32; M: 18	Danish	48.6 ± 11.1	28.9 ± 3.6	at risk of MetS	8	whole grain mix (ingredient: 179 g/day)	refined grain diet	≥ 5	FBG, FBI, HOMA-IR, HbA_1C_
Malik et al., 2019 [22]	crossover	112	NA	India	37.1 ± 9.4	28.1 ± 3.4	overweight	12	parboiled brown rice; total whole grain: 31.1 g/day	white rice; total whole grain: 15.7 g/day	1–2	FBG, FBI, HOMA-IR, HbA_1C_
Kuroda et al., 2019 [63]	parallel	I: 17; C: 17	F: 15; M: 19	Japan	I: 70.5 ± 0.9; C: 75.4 ± 1.1	I: 24.8 ± 0.6; C: 22.5 ± 0.5	healthy	104	100 g/d ultra-high hydrostatic pressurizing brown rice	100 g/d polished white rice	1–2	FBG, HbA_1C_
Hoevenaars et al., 2019 [62]	parallel	I: 25; C: 25	F: 31; M: 19	Netherlands	I: 28.0 ± 2.1; C: 27.6 ± 2.6	I: 47–69; C: 51–70	overweight and obese men and postmenopausal women	12	whole grain wheat cereals and ready-to-eat-cereals (ingredient: 98 g/day)	refined wheat	1–2	FBG, FBI, HOMA-IR
Kikuchi et al., 2018 [61]	parallel	I: 24; C: 25	F: 17; M: 32	Japan	I: 48.1 ± 1.6; C: 47.0 ± 1.7	I: 27.1 ± 0.7; C: 27.7 ± 0.48	healthy	12	whole grain wheat bread (ingredient: 100 g/day)	100 g/day refined wheat bread	1–2	FBG, FBI
Karl et al., 2017 [60]	parallel	I: 41; C: 40	F: 32; M: 49	USA	I: 55 ± 6; C: 54 ± 5	I: 25.7 ± 3.9; C: 25.7 ± 3.2	healthy	6	whole grain mix: (ingredient: 207 g/day)	refined grain-based diet; (ingredient: 0 g/day)	≥ 5	FBG, FBI, HOMA-IR
Nakayama et al., 2017 [23]	crossover	8	F: 4; M: 12	Japan	64.0 ± 8.8	25.7 ± 5.6	type 2 diabetes	8	277 g/day cooked glutinous brown rice	277 g/day cooked white rice	1–2	HbA_1C_
Kristensen et al., 2017 [59]	parallel	I: 81; C: 88	F: 169	France	I: 36.2 ± 10.1; C: 35.3 ± 8.7	I: 30.2 ± 1.9; C: 30.1 ± 2.0	overweight/obese	12	whole grain mix: 80 g/day	Refined grain	≥ 5	FBG, FBI, HbA_1C_
Kondo et al., 2017 [58]	parallel	I: 14; C: 14	F: 18; M: 10	Japan	I: 65.2 ± 8.7; C: 68.1 ± 6.8	I: 24.2 ± 3.5; C: 25.0 ± 3.7	type 2 diabetes	8	brown rice: for 10 out of 21 meals per week	white rice	1–2	FBG, FBI, HOMA-IR, HbA_1C_
Cooper et al., 2017 [24]	parallel	I: 35; C: 10	F: 25; M: 21	USA	I: 26.2 ± 1.0; C: 24.6 ± 1.6	I: 22.8 ± 0.5; C: 25.5 ± 2.1	healthy	6	6 servings/day whole grain products	refined grain	≥ 5	FBG
Vetrani et al., 2016 [57]	parallel	I: 26; C: 28	F: 24; M: 16	Italy	I: 57.2 ± 1.9; C: 58.4 ± 1.6	I: 32.1 ± 1.4; C: 31.5 ± 1.3	overweight/obese, MetS	12	whole grain mix (ingredient: 136 g/day)	refined grain	≥ 5	FBG, FBI, HOMA-IR
Kirwan et al., 2016 [56]	crossover	33	F: 27; M: 6	USA	39 ± 7	33.1 ± 4.3	overweight/obese	8	complete whole grain mix (ingredient : 93 g/day)	refined grain mix	≥ 5	FBG, FBI, HOMA-IR, HbA_1C_
Geng et al., 2016 [55]	parallel	I: 94; C: 97	F: 96; M: 95	China	I: 56.7 ± 6.8; C: 56.7 ± 6.7	I: 25.6 ± 3.0; C: 25.9 ± 2.8	hyperlipidemia	12	150 g/day pre-germinated brown rice	equivalent staple food products	1–2	FBG
Connolly et al., 2016 [54]	crossover	30	F: 19; M: 11	England	42	26.4 ± 5.7	at risk of cardio-metabolic disease	6	45 g/day of whole grain oat Granola	non-whole grain	1–2	FBG, FBI, HOMA-IR
Ampatzoglou et al., 2016 [53]	crossover	33	F: 21; M: 12	England	48.8 ± 1.1	27.9 ± 0.7	healthy	6	whole grain mix (ingredient: 168.4 g/day)	diet low in whole grain	≥ 5	FBG, FBI
Vitaglione et al., 2015 [52]	parallel	I: 36; C: 32	F: 45; M: 23	Italy	I: 40 ± 2; C: 37 ± 2	I: 30.0 ± 0.5; C: 29.5 ± 0.4	overweight/obese	8	62 g/day whole grain wheat biscuit	refined wheat products	1–2	FBG
Jackson et al., 2014 [51]	parallel	I: 25; C: 25	F: 25; M: 25	USA	I: 46.4 ± 5.9; C: 45.8 ± 6.0	I: 32.9 ± 3.5; C: 33.5 ± 4.0	Overweight/obese, at risk of metS	12	232 g/day whole grain products	refined grain	≥ 5	FBG, FBI, HOMA-IR
Bui et al., 2014 [50]	parallel	I: 30; C: 30	F: 60	Vietnam	I: 56.9 ± 5.8; C: 56.6 ± 5.0	I: 23.9 ± 3.0; C: 23.5 ± 3.2	pre-diabetes	16	pre-germinated brown rice (PGBR)	white rice	1–2	FBG
Wang et al., 2012 [49]	parallel	I: 29; C: 28	F:38; M:19	USA	I: 55 ± 9; C: 50 ± 9	I: 26.5 ± 3.0; C: 25.0 ± 2.2	pre-diabetes	12	brown rice as staple food	white rice	1–2	FBG, FBI, HbA_1C_, HOMA-IR
Mackay et al. a, 2012 [48]	crossover	14	F:4; M: 10	Canada	53 ± 6	26.5 ± 2.9	Normoglycemic/ normoinsulinemic	6	162.5 g/day whole grain wheat sourdough	168.8 g/day white bread	1–2	FBG
Mackay et al. b, 2012 [48]	crossover	13/14	F:4; M: 10	Canada	57 ± 7.4	35.7 ± 5.65	pre-diabetes	6	162.5 g/day whole grain wheat sourdough	168.8 g/day white bread	1–2	FBG
Kristensen et al., 2012 [47]	parallel	I: 38; C: 34	F: 72	Denmark	I: 59.1 ± 5.6; C: 60.3 ± 5.3	I: 30.0 ± 0.4; C: 30.4 ± 0.6	overweight/obese	12	150 g/day whole grain wheat bread/pasta/biscuits (ingredient: 105 g/day)	refined wheat	1–2	FBG, FBI, HOMA-IR, HbA_1C_
Zhang et al., 2011 [46]	parallel	I: 101; C: 101	F: 94; M: 108	China	I: 49.6 ± 6.7; C: 49.8 ± 7.1	I: 25.9 ± 3.4; C: 25.4 ± 2.5	diabete/at risk of diabetes	16	100 g/day brown rice	white rice	1–2	FBG, FBI, HOMA-IR, HbA_1C_
Tighe et al., 2010 [45]	parallel	I1: 73; I2:70; C: 63	F: 102; M: 104	England	I1: 51.6 ± 0.8; I2: 52.1 ± 0.9; C: 51.8 ± 0.8	I1: 28.0 ± 0.5; I2: 27.0 ± 0.4; C: 28.0 ± 0.5	healthy adults	12	I1: 3 servings of whole wheat products: 70–80 g whole grain bread + 30–40 g whole grain cereals; I2: 1 serving of whole wheat products and 2 servings of oats products	refined grain mix	≥ 5	FBG, HOMA-IR
Giacco et al., 2010 [44]	crossover	15	F:3 M: 12	Italy	54.5 ± 7.6	27.4 ± 3.0	healthy	3	whole grain wheat products	refined wheat products	≥ 5	FBG, FBI, HOMA-IR
Brownlee et al., 2010 [43]	parallel	I1: 85; I2: 81; C: 100	F: 133; M: 133	England	I1: 45.9 ± 10.1; I2: 45.7 ± 9.9; C: 45.6 ± 10	I1: 30.0 ± 3.7; I2: 30.3 ± 4.5; C: 30.0 ± 4.0	adults with BMI > 25	16	I1: 60 g/day whole grain products for 16 weeks; I2: 60 g/day whole grain products for 8 weeks followed by 120 g/day whole grain products for 8 weeks	consuming < 30 g/day whole grain products	≥ 5	FBG, FBI
Kim et al., 2008 [42]	parallel	I: 23; C: 24	F: 47	Korea	20–35	I: 27.57 ± 0.43; C: 27.27 ± 0.59	overweight women	6	mixture of brown rice and black rice 3 meals per day	white rice	1–2	FBG, FBI
Katcher et al., 2008 [41]	parallel	I: 24; C: 23	F: 23; M: 24	USA	I: 45.4 ± 8; C: 46.6 ± 9.7	I: 35.5 ± 4.1; C: 36.1 ± 4.9	obese adults with MetS	12	5 servings/day; obtain all of their grain servings from whole grains	<0.2 servings/day; avoid whole grain products	≥ 5	FBG, FBI
Andersson et al., 2007 [40]	crossover	30	F: 22; M: 8	Sweden	59 ± 5	28.3 ± 2.0	moderately overweight adults and/or with abdominal obesity	6	whole grain products (ingredient: 112 g/day)	refined grain	≥ 5	FBG, FBI
McIntosh et al., 2003 [39]	crossover	28	M: 28	Australia	40–65	30 ± 0.9	overweight men	4	products containing whole grain rye flour and wheat flour (ingredient: 88 g)	low-fiber refined cereal products	≥ 5	FBG, FBI
Li et al., 2003 [38]	crossover	10	F: 10	Japan	20.4 ± 1.3	19.2 ± 2.0	healthy	4	70% rice and 30% barley mixture	100% rice	1–2	FBG, HbA_1C_
Pins et al., 2002 [37]	parallel	I: 45; C: 43	F: 43; M: 45	USA	I: 48.7 ± 16.9; C: 46.4 ± 15.3	I: 31.2 ± 5.1; C: 30.6 ± 4.7	Men and women being treated for hypertension	12	60 g Quaker oatmeal + 77 g Quaker oat squares	65 g Malt-O-Meal hot wheat cereal + 81 g Kellogg’s crispix	1–2	FBG
Pereira et al., 2002 [36]	crossover	11	F:6; M:5	USA	41.6 ± 2.67	30.2 ± 1.01	overweight and obese hyperinsulinemic adults.	6	whole grain mix (ingredient: 356 g/day)	refined grain mix	≥ 5	FBG, FBI, HOMA-IR
Hawrysh et al., 1998 [35]	crossover	11	M: 11	Canada	51 ± 6.5	27.4 ± 0.1	type 2 diabetes patients	12	83 g/day waxy hulless barley bread	white bread	1–2	FBG, FBI, HbA_1C_

^a^C, control group; FBG, fasting blood glucose; F, female; FBI, fasting blood insulin; HbA_1C_, glycemic hemoglobin; HOMA-IR, homeostasis model assessment of insulin resistance; I, intervention group; I1-I3, different intervention groups; M, male; MetS, metabolic syndrome

^b^Age and baseline BMI was described as mean (± SD) or range

Out of 37 RCTs, 14 studies were performed in Asia [22, 23, 38, 42, 46, 50, 55, 58, 61, 63, 65–68], 10 in the North America [24, 35–37, 41, 48, 49, 51, 56, 60], 12 in Europe [40, 43–45, 47, 52–54, 57, 59, 62, 64], and 1 in Australia [39]. Intervention duration was from 3 weeks to 2 years, and whole grain ingredient consumption ranged from 22.5 g/day to 207 g/day. Some RCTs had high-performance bias because they failed to implement blind intervention on subjects, and no other significant sources of bias were observed (Supplemental Table 2).

### The association of whole grains and type 2 diabetes

**Fig. 2:**
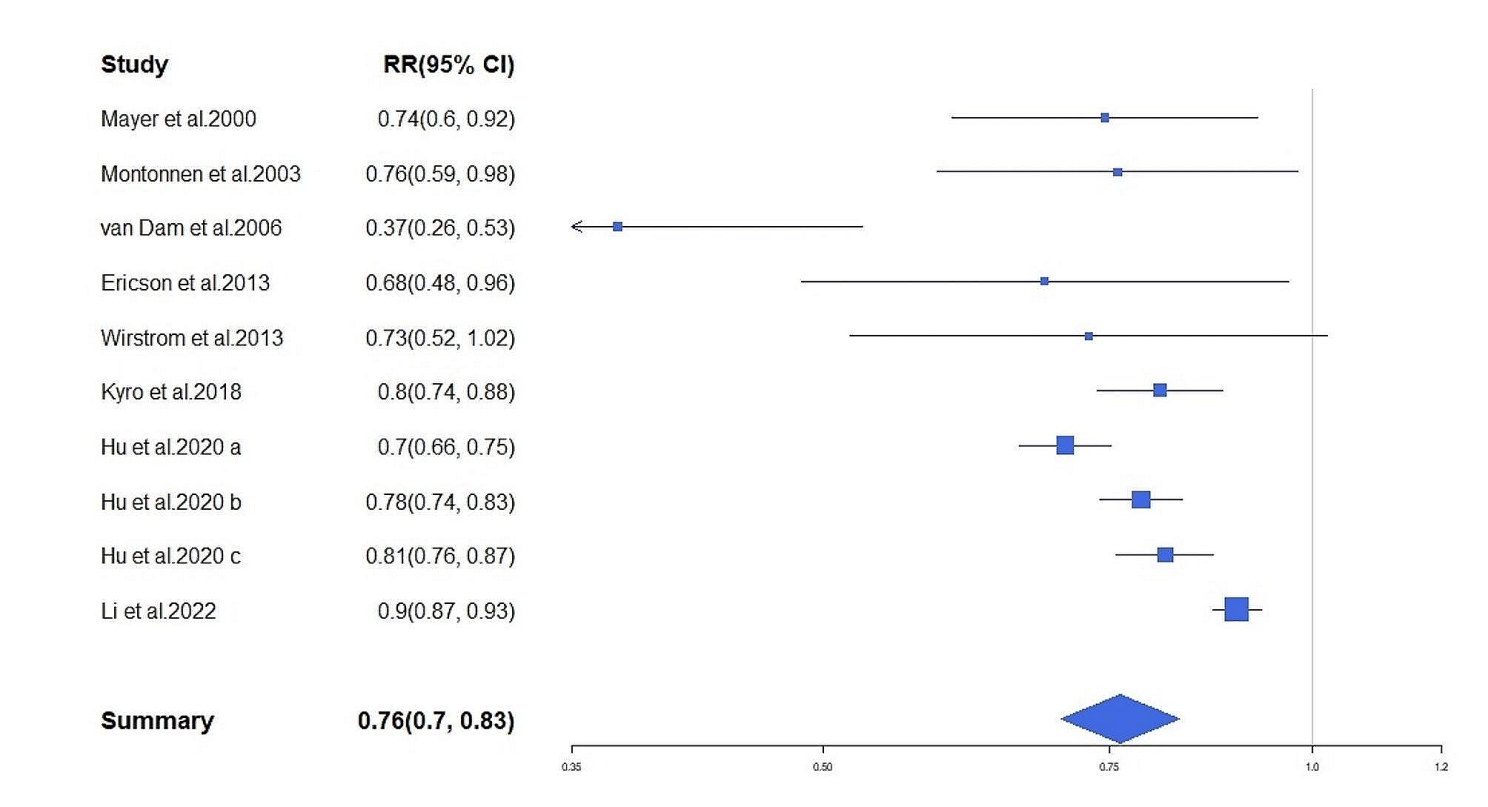
Forest plot for the effects of whole grains (50 g/day whole grain ingredient) on the risk of type 2 diabetes in adults. The area of each square is proportional to the inverse of the variance of the risk ratio. Horizontal lines represent 95% of CIs. The X-axis scale is logarithmically transformed

Ten cohort studies were included in the analysis of whole grains and type 2 diabetes risk, in which 47,023 diabetic cases were reported among 473,019 participants. The summary RR for 50 g/day intake of whole grain ingredient was 0.761 (95% CI: 0.700 to 0.828, *I*^*2*^ = 72.39%, *P* < 0.001, Fig. 2). According to the Begg’s test (Kendall’s tau = -0.20, *P* = 0.48) and the visual inspection of the funnel plot (Supplemental Fig. 1), no evidence of publication bias was found. A sensitivity analysis revealed that the overall estimate did not depend on any single study.

**Fig. 3:**
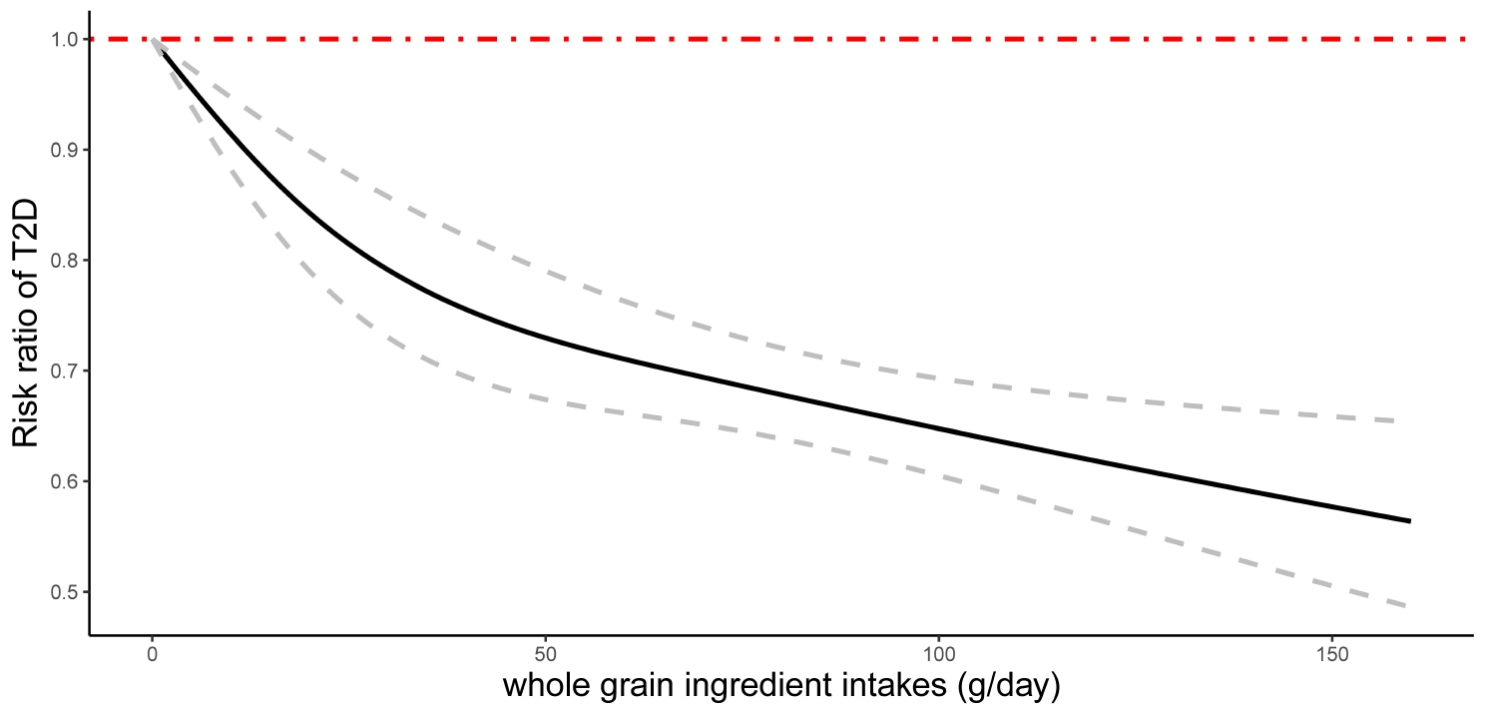
Nonlinear dose-response relationships between whole grain ingredient intake and the risk of type 2 diabetes in adults

Dose-response analyses indicated that the risk of type 2 diabetes was dose-dependent (β = -0.0052 (g/day)^−1^; 95% CI: -0.0067 to -0.0037). A nonlinear, inverse association (*P*_*non−linearity*_ = 0.01) was observed between the whole grain ingredient intake and type 2 diabetes occurrence (Fig. 3), with a reduction in risk lower than 50 g/day and the association was attenuated for higher values. Given the limited number of cohort studies, the subgroup analysis was not performed.

### The association of whole grains and fasting blood glucose

**Fig. 4:**
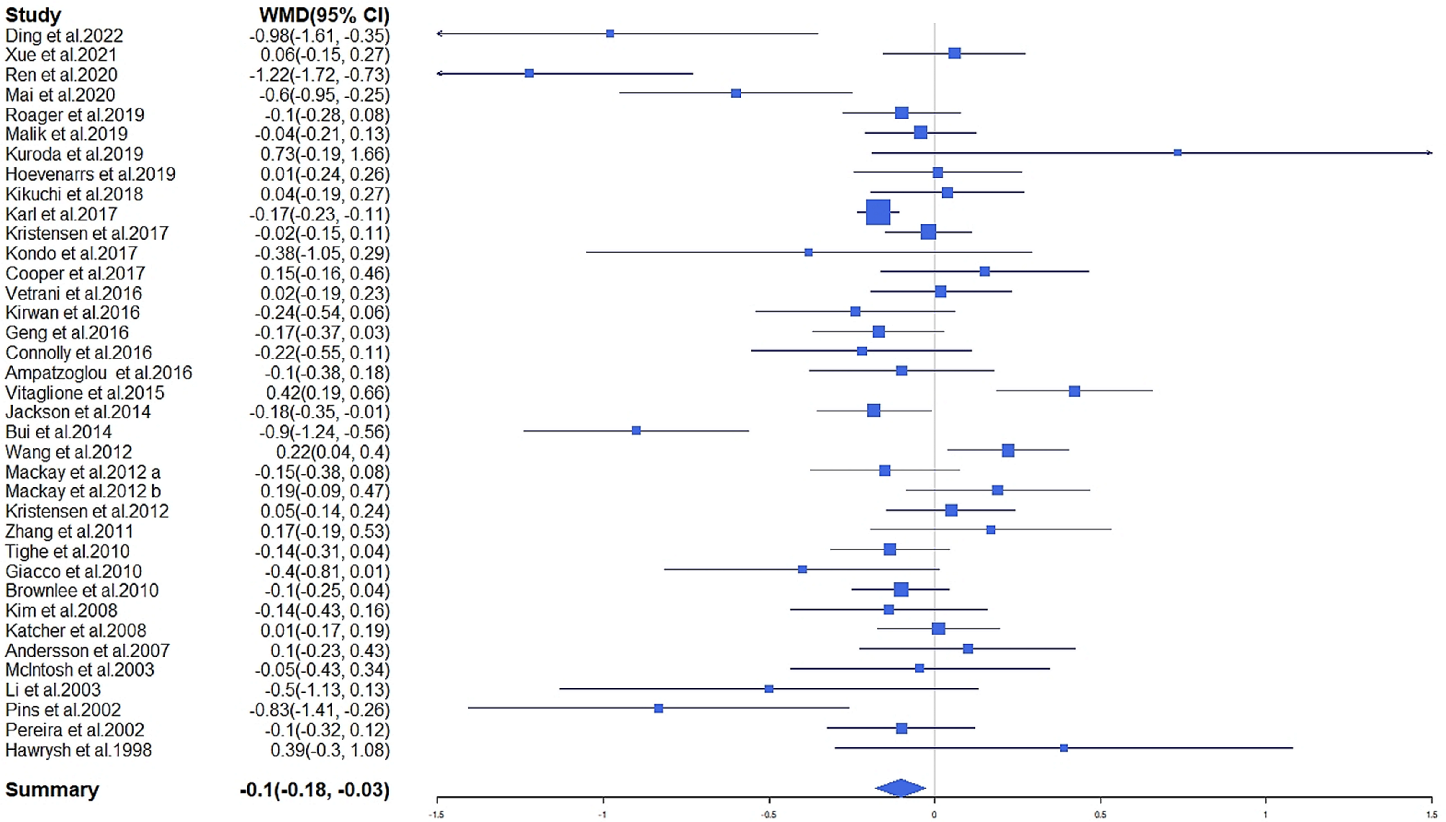
Forest plot for the effects of whole grains on fasting blood glucose in adults, expressed as mean differences between intervention and control groups. The area of each square is proportional to the inverse of the variance of the weighted mean difference. Horizontal lines represent 95% of Cis

In total, 37 RCTs accounting for 3116 subjects reported FBG changes for the whole grain interventions. FBG after intervention was significantly lower compared with the control groups (MD = -0.103 mmol/L, 95% CI: -0.178 to -0.028; *I*^*2*^ = 72.99%, *P* < 0.01; Fig. 4). According to Begg’s test (Kendall’s tau = -0.12, *P* = 0.30) and the visual inspection of the funnel plot (Supplemental Fig. 2), no evidence of publication bias was found. A sensitivity analysis revealed that the overall estimate did not depend on any single study. Replacing the baseline to follow-up correlation with either 0.5 or 0.9, the relationship between FBG and the whole grain intervention did not change (MD = -0.091 mmol/L, 95% CI: -0.162 to-0.020; *I*^*2*^ = 61.37%, *P* < 0.01; MD = -0.124 mmol/L, 95% CI: -0.208 to-0.004; *I*^*2*^ = 88.02%, *P* < 0.01, respectively). Replacing studies with the most extended follow-up years or the latest data to shorter and earlier ones in studies based on the same group of participants, the relationship between FBG and the whole grain intervention did not change.

**Fig. 5:**
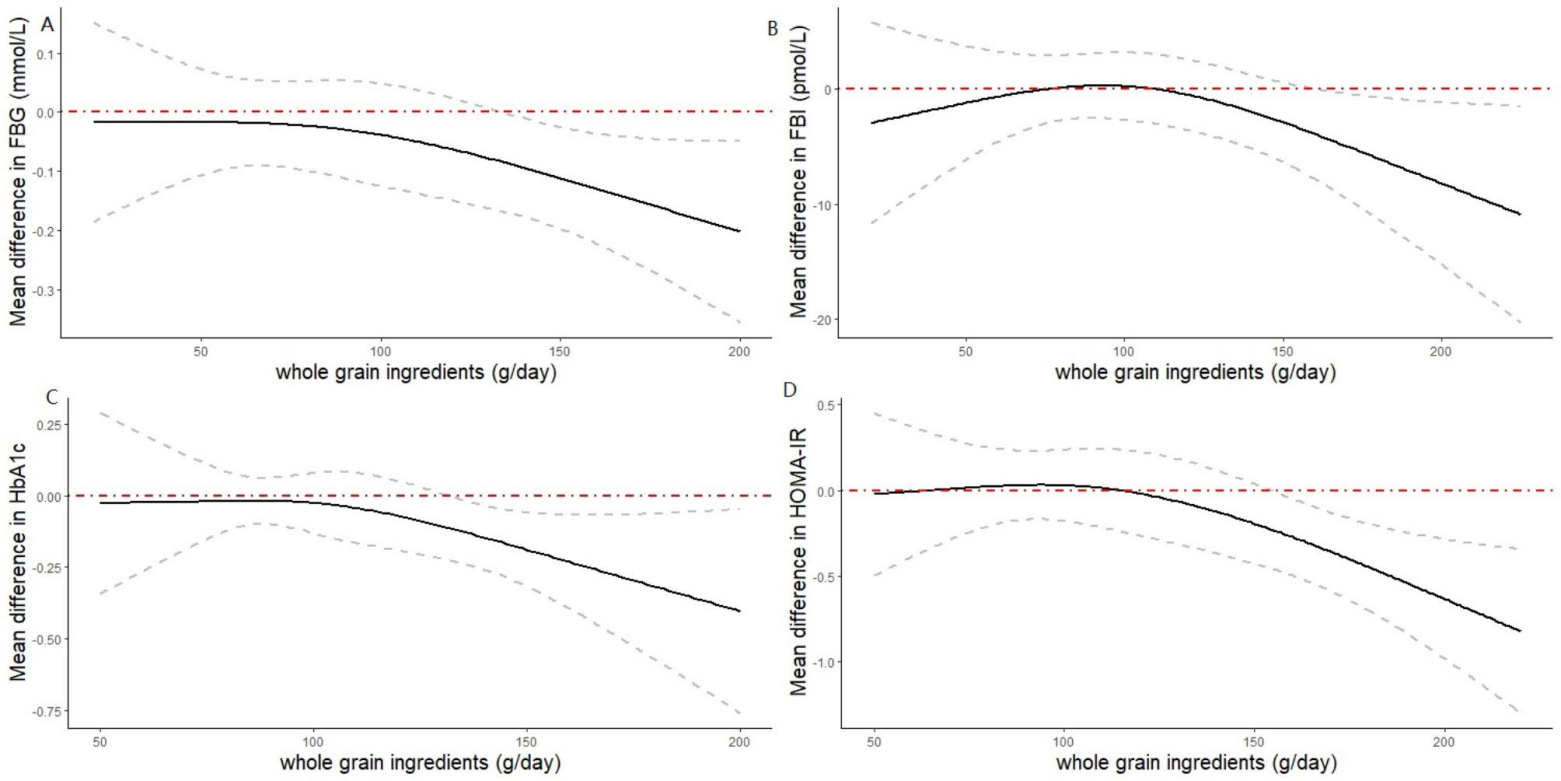
Nonlinear dose-response relationships between whole grain ingredients and mean differences in glycemic and insulin biomarkers in adults. (**a**) Fasting blood glucose (FBG). (**b**) Fasting blood insulin (FBI). (**c**) Glycated hemoglobin (HbA_1C_). (**d**) Homeostatic model assessment for insulin resistance (HOMA-IR)

In subgroup analysis (Table 3), we found that whole grain product variety, whole grain types and health status contributed to the between-study heterogeneity. Significant reductions in FBG were observed in studies conducted with ≥ 5 whole grain products (MD = -0.101 mmol/L; 95% CI: -0.154 to -0.048; *I*^*2*^ = 19.41%, *P* < 0.001), those intervened with mixed whole grain (MD = -0.092 mmol/L; 95% CI: -0.148 to -0.035; *I*^*2*^ = 22.95%, *P* = 0.03), and those intervened with brown rice (MD = -0.305 mmol/L; 95% CI: -0.564 to -0.045; *I*^*2*^ = 86.66%, *P* = 0.02). We observed a marginally significant effect in studies conducted with 1–2 whole grain products (MD = -0.144 mmol/L; 95% CI: -0.287 to -0.001; *I*^*2*^ = 80.60%, *P* = 0.048), but no significant effect was observed in studies conducted with wheat (MD = 0.019 mmol/L; 95% CI: -0.130 to 0.168; *I*^*2*^ = 68.27%, *P* = 0.80) and other whole grain types (MD = -0.164 mmol/L; 95% CI: -0.434 to 0.106; *I*^*2*^ = 59.92%, *P* = 0.23). We also observed significant reductions in FBG in studies with low risk (MD = -0.153 mmol/L; 95% CI: -0.254 to -0.053; *I*^*2*^ = 77.37%, *P* < 0.01), compared to those with uncertain (MD = -0.091 mmol/L; 95% CI: -0.189 to 0.007; *I*^*2*^ = 24.51%, *P* = 0.07) and high risk (MD = -0.082 mmol/L; 95% CI: -0.059 to 0.223; *I*^*2*^ = 37.73%, *P* = 0.12). Furthermore, linear regression indicated that the FBG improvement was dose-dependent (β=-0.0011 mmol/L*(g/day)^−1^; 95% CI: -0.0021 to -0.0001, *P* = 0.04). We found a significant non-linear association between whole grain and FBG (Fig. 5a; *P*_*non−linearity*_ = 0.04), and greater reduction was demonstrated at doses more than 150 g/day.

**Table 3:** Subgroup analysis on the effects of whole grains on fasting blood glucose, glycemic hemoglobin and homeostatic model assessment for insulin resistance in adults

	Subgroup	FBG (mmol/L)	HbA_1C_ (mmol/mol)	HOMA-IR
Study design	parallel	-0.116(-0.217, -0.014)* *N* = 24, I^2^ = 80.85	0.039(-0.334,0.412) *N* = 8, I^2^ = 0	-0.118(-0.308, 0.071) *N* = 11, I^2^ = 27.39
	crossover	-0.086(-0.165, -0.006)* *N* = 13, I^2^ = 8.46	-1.889(-3.519,-0.259)* *N* = 5, I^2^ = 72.61	-0.307(-0.766, 0.152) *N* = 4, I^2^ = 46.46
Intervention duration	≥ 12w	-0.097(-0.199, 0.006) *N* = 20, I^2^ = 73.83	0.041(-0.331,0.413) *N* = 8, I^2^ = 0	-0.112(-0.338, 0.114) *N* = 9, I^2^ = 41.07
	< 12w	-0.115(-0.233, 0.004) *N* = 17, I^2^ = 71.89	-2.018(-3.683,-0.353)* *N* = 5, I^2^ = 71.56	-0.281(-0.564, 0.001) *N* = 6, I^2^ = 14.75
Health status	unhealthy	-0.219(-0.409, -0.028)* *N* = 14, I^2^ = 84.14	-1.339(-2.598,-0.080)* *N* = 7, I^2^ = 71.58	-0.208(-0.453, 0.037) *N* = 7, I^2^ = 30.17
	generally healthy	-0.061(-0.136, 0.014) *N* = 22, I^2^ = 58.76	0.041(-0.369,0.452) *N* = 6, I^2^ = 0	-0.123(-0.397, 0.151) *N* = 8, I^2^ = 42
Baseline mean of BMI	BMI < 30 kg/m^2^	-0.102(-0.209, 0.005) *N* = 23, I^2^ = 76.01	-1.027(-2.036,-0.017)* *N* = 10, I^2^ = 69.46	-0.142(-0.349, 0.065) *N* = 10, I^2^ = 26.83
	BMI ≥ 30 kg/m^2^	-0.059(-0.143, 0.024) *N* = 11, I^2^ = 39.98	0.062(-0.38,0.505) *N* = 3, I^2^ = 0	-0.222(-0.608, 0.164) *N* = 5, I^2^ = 53.7
Baseline mean of age	age < 50	-0.098(-0.199, 0.003) *N* = 18, I^2^ = 69.85	-0.423(-1.071,0.225) *N* = 6, I^2^ = 33.75	-0.103(-0.441, 0.236) *N* = 6, I^2^ = 54.9
	age ≥ 50	-0.121(-0.199, 0.003) *N* = 17, I^2^ = 77.55	-0.866(-2.177,0.446) *N* = 7, I^2^ = 77.02	-0.198(-0.405, 0.01) *N* = 9, I^2^ = 17.24
Baseline mean of triglycerides	TG ≥ 1.7 mmol/L	-0.364(-0.737, 0.008) *N* = 6, I^2^ = 86.44	NA	-0.347(-0.809, 0.116) *N* = 4, I^2^ = 56.94
	TG < 1.7 mmol/L	-0.061(-0.13, 0.009) *N* = 8, I^2^ = 10.2	-0.198(-0.674,0.277) *N* = 5, I^2^ = 26.34	0.062(-0.148, 0.272) *N* = 6, I^2^ = 0
Whole grain products variety	1–2	-0.144(-0.287, -0.001)* *N* = 23, I^2^ = 80.60	-0.836(-1.818,0.147) *N* = 10, I^2^ = 68.06	-0.120(-0.413, 0.174) *N* = 7, I^2^ = 54.72
	≥ 5	-0.101(-0.154, -0.048)*** *N* = 14, I^2^ = 19.41	-0.414(-1.344,0.516) *N* = 3, I^2^ = 61.71	-0.229(-0.440, -0.019)* *N* = 8, I^2^ = 0
Whole grain types	Rice	-0.305(-0.564, -0.045)* *N* = 11, I^2^ = 86.66	-1.123(-2.922,0.675) *N* = 6, I^2^ = 78.87	-0.324(-0.729, 0.082) *N* = 4, I^2^ = 57.44
	Wheat	0.019(-0.130, 0.168) *N* = 8, I^2^ = 68.27	NA	-0.005(-0.267, 0.258) *N* = 4, I^2^ = 0
	Others	-0.164(-0.434, 0.106) *N* = 6, I^2^ = 59.92	-0.439(-1.566,0.689) *N* = 3, I^2^ = 0	NA
	Mix	-0.092(-0.148, -0.035)** *N* = 12, I^2^ = 22.95	-0.414(-1.344,0.516) *N* = 3, I^2^ = 61.71	-0.261(-0.540, 0.019) *N* = 6, I^2^ = 22.57
Study quality	Low risk	-0.153(-0.254,- 0.053)** *N* = 25, I^2^ = 77.37	-0.424(-0.899, 0.051) *N* = 7, I^2^ = 0	-0.089(-0.251, 0.073) *N* = 12, I^2^ = 19.23
	Uncertain	-0.091(-0.189, 0.007) *N* = 7, I^2^ = 24.51	-0.151(-0.429, 0.127) *N* = 3, I^2^ = 90.43	NA
	High risk	-0.082(-0.059, 0.223) *N* = 5, I^2^ = 37.73	0.170(-1.263 1.639) *N* = 3, I^2^ = 34.18	NA

***: *P* < 0.001; **: *P* < 0.01; *: *P* < 0.05

^a^FBG: fasting blood glucose; HbA_1C_: glycemic hemoglobin; HOMA-IR: homeostatic model assessment for insulin resistance

### The association of whole grains and fasting blood insulin

In total, 25 RCTs accounting for 2142 subjects reported fasting blood insulin for the whole grain interventions. There was no significant reduction in fasting blood insulin after the whole grain intervention (MD = -1.324 pmol/L, 95% CI: -3.611 to 0.963; *I*^*2*^ = 0% *P* = 0.26; Supplemental Fig. 3). No evidence of publication bias was found (Supplemental Fig. 4). A sensitivity analysis revealed that the overall estimate did not depend on any single study. Linear regression showed a negative but insignificant relationship between whole grain ingredient intake and FBI changes (β = -0.041 pmol/L*(g/day)^−1^; 95% CI: -0.097 to 0.015, *P* = 0.14). When the restricted spline model was implicated (Fig. 5b; *P*_*non-linearity*_ =0.12), we observed an inverted U-shaped curve.

### The effects of whole grains on glycated hemoglobin

In total, 13 RCTs accounting for 1043 subjects reported HbA_1C_ for the whole grain interventions. Pooling these effect sizes (Supplemental Fig. 5), we found a modest effect of whole grain consumption on HbA_1C_ (MD = -0.662 mmol/mol (-0.06%), 95% CI: -1.335 to 0.010; *I*^*2*^ = 64.55%, *P* = 0.053). According to Begg’s test (Kendall’s tau = -0.44, *P* = 0.04) and the funnel plot (Supplemental Fig. 6), no evidence of publication bias was found. A sensitivity analysis revealed the influence of a study by Kristensen et al. [59], with the results demonstrating some beneficial effects of whole grain on HbA_1C_ (MD = -0.820 mmol/mol (-0.08%), 95% CI: -1.585 to -0.051; *I*^*2*^ = 62.92%, *P* = 0.04). It was worth noting that in that study, a non-compliance of 60% was reported.

In subgroup analysis (Table 3), study design, intervention duration and health status contributed to the between-study heterogeneity. We observed a significant effect of whole grain on HbA_1C_ in the studies with crossover design (MD = -1.889 mmol/mol (-0.17%); 95% CI: -3.519 to -0.259; *I*^*2*^ = 72.61%, *P* = 0.02), those conducted in unhealthy participants (MD = -1.339 mmol/mol (-0.12%); 95% CI: -2.598 to -0.080; *I*^*2*^ = 71.58%, *P* = 0.04) and those with duration < 12 w (MD = -2.018 mmol/mol (-0.18%); 95% CI: -3.683 to -0.353; *I*^*2*^ = 71.56%, *P* = 0.02). We did not observe any significant effect in the studies with parallel design (MD = 0.053 mmol/mol (0.01%); 95% CI: -0.322 to 0.428; *I*^*2*^ = 0%, *P* = 0.78), those conducted in generally healthy participants (MD = 0.041 mmol/mol (0.004%); 95% CI: -0.369 to 0.452; *I*^*2*^ = 0%, *P* = 0.83) and those with duration ≥ 12 w (MD = 0.055 mmol/mol (0.01%); 95% CI: -0.319 to 0.428; *I*^*2*^ = 0%, *P* = 0.78). Linear regression indicated that the HbA_1C_ improvement was dose-dependent (β = -0.027 mmol/mol*(g/day)^−1^; 95% CI: -0.051 to -0.003, *P* = 0.02). We discovered a significant non-linear association between whole grain and HbA_1C_, (Fig. 5c; *P*_*non−linearity*_ = 0.04), and greater reduction was observed at doses more than 120 g/day.

### The association of whole grains and HOMA-IR

In total, 15 RCTs accounting for 1254 subjects reported HOMA-IR for whole grain intervention. Pooling these effect sizes (Supplemental Fig. 7), we observed a modest effect of whole grain consumption on HOMA-IR (MD = -0.164, 95% CI: -0.342 to 0.013; *I*^*2*^ = 33.38%, *P* = 0.07). According to Begg’s test (Kendall’s tau = -0.20, *P* = 0.32) and the funnel plot (Supplemental Fig. 8), no evidence of publication bias was found. A sensitivity analysis revealed the influence of a study by Xue et al. [67], with the results demonstrating some beneficial effects of whole grain on HOMA-IR (MD = -0.203, 95% CI: -0.373 to -0.032; *I*^*2*^ = 23.24%, *P* = 0.02). However, in that study, poor compliance in the whole grain group was reported by the assessment of plasma alkylresorcinol.

In subgroup analysis (Table 3), we found that whole grain product variety could contribute to the between-study heterogeneity. We observed a significant effect on HOMA-IR in studies conducted with ≥ 5 whole grain products (MD = -0.229; 95% CI: -0.440 to -0.019; *I*^*2*^ = 0%, *P* = 0.03) while we did not observe any significant effect in studies that conducted with 1–2 whole grain products (MD = -0.120; 95% CI: -0.413 to 0.174; *I*^*2*^ = 54.72%, *P* = 0.42). Linear regression indicated that the HOMA-IR improvement was dose-dependent (β = -0.0049 (g/day)^−1^; 95% CI: -0.171 to -0.031, *P* = 0.01). We observed a significant non-linear relationship between whole grain consumption and HOMA-IR (Fig. 5d; P_*non-linearity*_ < 0.001), with a greater reduction observed at doses exceeding 150 g/day.

## Discussion

### Summary of findings

Our systematic meta-analyses demonstrated the probable benefits of whole grain consumption for glycemic control. The summary from prospective cohort studies indicated that a 50 g/day intake of whole grain ingredients would protect against type 2 diabetes with a 25% reduction in the relative risks. Such findings were partially supported by RCTs, which indicated that an all-around improvement of glycemic markers could be obtained by an intake of 150 g/day of whole grain ingredients. Specifically, whole grain intake could improve fasting blood glucose, with modest effects on glycated hemoglobin and insulin resistance. However, there was no significant difference in fasting blood insulin. Furthermore, a combination of whole grains should be encouraged because participants who intervened with various whole grain products achieved greater glycemic control in RCTs.

### Comparisons with the existing literature

Evaluating evidence for nutritional guidance across multiple study designs might be challenging. The impacts of such evaluations should be collected at a system level from prospective cohort studies of clinical endpoints and controlled trials of intermediate pathways [69]. Previous prospective cohort studies provided assessments of whole grain consumption over the long term (from 6 to 40 years) in a large number of participants (*n* = 473,019), providing sufficient time for the etiologies of diet-related chronic disease. However, these cohort studies were carried out on participants from limited regions of the world, namely America and the Nordics, who mainly consumed whole grain wheat with narrow intake ranges. Furthermore, the cohort studies on whole grains were particularly susceptible to confounding. Whole grain intake was associated with a healthy lifestyle, such as low BMI, frequent participation in sports, and moderate alcohol intake [70, 71]. Moreover, the lack of recognition of whole grain ingredients in foods might bring a bias in self-reporting whole grain intake in dietary assessments [72]. On the contrary, RCTs were preferable to minimize confounding effects and were more generalizable. For example, this analysis included 37 RCTs conducted in 14 countries, and only 11 of 37 were on American and Nordics. In particular, 14 RCTs were conducted on Asians with high rice consumption. However, RCTs were challenging to detect notable effects in a short duration (mainly 3 to 16 weeks) among a relatively small number of participants (*n* = 3116). With increased intervention durations, more significant improvements in glycemic control could be observed. Nevertheless, in our study, prospective cohort studies showed a reduction in the incidence of type 2 diabetes, while RCTs demonstrated various improvements in glycemic biomarkers.

However, the results of previous meta-analyses and ours did not always corroborate. For example, Li et al. indicated significant effects of whole grains on FBG, FBI, HbA_1C,_ and HOMA-IR. At the same time, Marventano et al. observed no significant results in those measures [18, 19]. One of the possible reasons for such controversial results could be that Li et al. included 8 studies with multiple arms conducted in the same population and were analyzed in meta-analysis as separate studies, which could bring a high risk of unit-of-analysis error. Furthermore, Marventano et al. excluded some crucial sources of whole grains (e.g., brown rice) in their meta-analysis. Moreover, these two meta-analyses both included studies with crossover design but reported the outcomes in two distinct phases.

### What the study adds to the existing literature

In our meta-analysis, given prominence by the guidance of the Cochrane Handbook [73], we selected the publications with the longest follow-up years or the latest data from the publications based on the same group of participants and created single pair-wise comparisons combined with multiple arms in studies, and excluded studies with crossover design which reported the outcomes by two distinct phases [74, 75]. Also, we did not include pseudo-grain (e.g. buckwheat, Quinoa, amaranth) because people rarely eat whole grain products made by pseudo-cereal grains [72]. We hope these methodological developments could greatly help the current study, further supporting our findings that whole grain intake could significantly improve fasting blood glucose and insulin sensitivity.

Another concern for evaluating evidence from both cohort studies and interventions was the heterogeneity in the meta-analysis, which existed in both types of studies on whole grains. While this heterogeneity could not be adequately assessed for the cohorts as there were too few cohort studies, subgroup analyses of the RCTs identified potential sources of heterogeneity. A significant reduction in FBG and HOMA-IR was found in the studies with various whole grain products while whole grain products only had a borderline significant effect on FBG. In our study, participants provided with ≥ 5 whole grain products were characterized to have a diet of various whole grain products, in which participants consumed whole grain products (e.g., bread, breakfast cereal, pasta, rice, couscous, bars, snacks, et al.) in an ad libitum manner or according to menus. It was reported that whole grains could reduce hunger and increase fullness compared to refined grains, which brought a high risk of non-compliance in whole grain intervention studies [76]. Increases in whole grain choices could probably improve compliance. Moreover, the diversity in nutrient compositions of each whole grain would also lead to differential effects on glycemic control. In our study, mixed whole grains significantly reduced FBG. As for single whole grain, brown rice, rather than whole grain wheat, had a significant effect on FBG, while insufficient evidence was available for the effects of oats, barley and rye. Wheat and rye primarily contain non-viscous and poorly fermentable fibers, while oats and barley are rich in β-glucan, which is viscous and fermentable [76]. There was apparent evidence from RCTs that β-glucan could reduce glycemic and insulinemic responses by slowing the digestion of carbohydrates and promoting the growth of probiotics, while the observations from non-viscous fibers were not as apparent [77, 78]. Besides, numerous studies have demonstrated that the phenolic compounds vary among the husks of wheat, barley, oats and rice [79]. Brown rice and barley are rich in hydroxybenzoic acids, while wheat and oats are rich in hydroxycinnamic acids [80]. Also, brown rice is a good source of γ-oryzanol, phytosterols, and aminobutyric acid [81]. These bioactive compounds might help hamper oxidative stress, reduce subclinical inflammation, and inhibit α-amylase and α-glucosidase activities [11, 82]. Thus, such diversities in nutrient combinations among whole grains could synergistically impact the study outcomes. It was further argued that similar dietary advice on whole grains should be applied for both the prevention and management of type 2 diabetes [69]. Our results from the subgroup analyses of RCTs partially supported this claim. We found that participants with or at risk of metabolic diseases achieved better results on FBG and HbA_1C_ compared with generally healthy participants. We also included factors such as baseline age and levels of triglycerides in subgroup analysis. However, the results revealed that those factors were not the primary sources of heterogeneity. All in all, our analyses of RCTs, compliant with the prospective cohort studies, suggested that the effectiveness of whole grains might vary by different types and that consuming multiple whole grain products might exert mutually reinforcing benefits on glycemic control.

### Implications of our findings for policymakers

Despite the clear indications from evidence-based analyses that whole grains could improve glycemic responses in some form, dietary guidelines targeting individual consumption are lacking to help stem the emerging pandemic of type 2 diabetes. The current statements aiming at promoting whole grain consumption, such as “choosing whole grain varieties whenever you can” from the Eat Well guide from the UK government [7] and “make at least half of grains whole grains” from dietary guidelines for Americans (DGA) [6], are commonly generic and vague, and only a limited number of countries and organizations proposed quantitative recommendations ranged 45–232 g/day [9]. For instance, the EAT-Lancet Commission on Healthy Diets from Sustainable Food Systems recommended 232 g/day of whole grains to maintain energy intake. DGA recommended 48 g/day of whole grains to substitute half of daily grains [6, 8]. Indeed, the previous data might be insufficient to make separate estimations on the effects of doses, nutrition components, and processing methods. As far as we know, the dose-response meta-analyses on reducing type 2 diabetes risk focused mainly on observational studies [12, 13] but not on RCTs, representing a greater variety of intervention doses and populations. The lack of globally recognized methods of ingredient calculations in whole grain products has also made comparisons between studies challenging [72]. With the latest boom of whole grain studies and improved methodology, we should come closer to the ultimate answer: how much whole grains should be consumed to reduce type 2 diabetes risks. In this study, we conducted dose-response meta-analyses on both cohort studies and RCTs and quantified the intakes by whole grain ingredients rather than whole products. It was conservatively estimated that whole grain products contained, on average, 51% of whole grain ingredients because most countries claimed that whole grain products should contain 50 ∼ 100% whole grain ingredients. Our results from cohort studies suggested that any increase in whole grain intakes would benefit the prevention of type 2 diabetes. However, the evidence from RCTs indicated that the preventive efficacy of whole grain ingredients on glycemic control could only be obtained at doses > 150 g/day. Moreover, dose-response analysis indicated restricted generalization and residual confounding in cohort studies. Therefore, from our perspective, a feasible recommendation in type 2 diabetes prevention could be the dose ranges where cohort studies and RCTs aligned. To our knowledge, few studies reported adverse effects of high whole grain intakes on health outcomes. Collectively, advice on consuming > 150 g/day whole grain ingredients could be a low-risk public health strategy for general populations, which would be affordable and wide-reaching for country-specific cultural diets.

### Strengths and weaknesses

The present study had several strengths. Arguably, the most important one was the parallel evaluations of whole grains in both prospective cohort studies and RCTs. The former approach involved the examination of the effects on type 2 diabetes, and the latter accessed the biomarkers of type 2 diabetes as outcomes. Although the dose-response effects of whole grains differed between the type 2 diabetes incidences and measures of glycemic control, aggregated data from RCTs and cohort studies could provide a solid evidence base for updating whole grain recommendations. Secondly, all the cohort studies included had a prospective design, reducing the risks of recall and selection bias. Thirdly, whole grains were proven effective in lowering fasting blood glucose and HbA_1C_ in the studies with crossover design, which met the gold standard for randomized controlled trials. However, several limitations should also be acknowledged. Firstly, we extrapolated the whole grain ingredients by the assumption that whole grain foods contained 51% of whole grain ingredients on average. It would slightly underestimate the amount of whole grains, probably leading to underestimating the recommended whole grain intake. Future applications of a standardized methodology to calculate whole grain intake are needed. Secondly, the subgroup analyses were less reliable in the subgroups with smaller numbers of RCTs. Thirdly, testing the publication bias for limited cohort studies might be hard. In addition, treating glycemic control as the secondary outcome, as well as the small sample sizes of RCTs, might also contribute to the heterogeneity of this meta-analysis.

## Conclusion

This study suggests a significant beneficial effect of whole grain consumption on glycemic control and reducing type 2 diabetes risks. Consuming more than 150 g of whole grain ingredients daily would be highly recommended to prevent type 2 diabetes in general populations. This information provides a more comprehensive evidence base for the revision of dietary recommendations on whole grains and contributes to improving public health strategies targeting type 2 diabetes prevention and management.

### Electronic supplementary material

Below is the link to the electronic supplementary material.

Supplementary Material 1

Supplementary Material 2

Supplementary Material 3

## Data Availability

All data generated or analyzed during this study are included in this published article (and its supplementary information files).

## Abbreviations

FBG: fasting blood glucose
FBI: fasting blood insulin
MD: mean difference
MetS: metabolic syndrome
TG: triglycerides
