# Supplementary material for: Effects of whole grains on glycemic control: a systematic review and dose-response meta-analysis of prospective cohort studies and randomized controlled trials
Source: Nutr J. 2024 Apr 25;23:47. doi: 10.1186/s12937-024-00952-2 (PMC11044462; doi:10.1186/s12937-024-00952-2)
Supplement: Supplementary file 3 — Supplementary Material 3 [file 12937_2024_952_MOESM3_ESM.docx]

# Effects of whole grains on glycemic control: a systematic review and dose-response meta-analysis of prospective cohort studies and randomized controlled trials

Tao Ying^1^^, Jianheng Zheng^2^^, Juntao Kan^2^, Wenyun Li^1^, Kun Xue^1^, Jun Du^2^, Yuwei Liu^1^*, Gengsheng He^1^*

1. *School of Public Health, Key Laboratory of Public Health Safety of the Ministry of Education, Fudan University, Shanghai 200032, China.*
2. *Nutrilite Health Institute, Shanghai, China*

^Both authors contribute equally.

* Both authors are corresponding authors.

*Yuwei Liu, School of Public Health, Key Laboratory of Public Health Safety of the Ministry of Education, Fudan University, 130 Dong’an Road, Shanghai 200032, China

Phone: 86-21-54237229, Fax: 86-21-54237271

E-mail: [ywliu@fudan.edu.cn](mailto:ywliu@fudan.edu.cn)

*Gengsheng He, School of Public Health, Key Laboratory of Public Health Safety of the Ministry of Education, Fudan University, 130 Dong’an Road, Shanghai 200032, China

Phone: 86-21-54237271, Fax: 86-21-54237271

E-mail: [gshe@shmu.edu.cn](mailto:gshe@shmu.edu.cn)

# Supplementary methods:

## Search strategy

We searched PubMed, Clarivate Web of Science, and Cochrane Library up to December 2021, using the following search terms: (“whole grain” OR “whole-grain” OR “whole grains” OR “whole meal” OR “whole wheat” OR “whole kernel” OR “grains” OR “cereals” OR “wheat” OR “oat” OR “brown rice” OR “barley” OR “rye”) and (“diabetes” OR “blood sugar” OR “blood glucose” OR “fasting plasma glucose” OR “FBG” OR “insulin” OR “glycated hemoglobin” OR “HbA1c” OR “insulin resistance” OR “HOMA-IR”). The search was restricted to studies published in English.

## Selection of the studies

The inclusion criteria were determined in accordance with the populations, interventions, comparators, outcomes, and study designs (PICOS) guideline (**Supplemental Table 1**):

1. Studies that investigated the effect of whole grains on the risk of type 2 diabetes (for prospective cohort studies) and intermediate glycemic biomarkers (for RCTs), including fasting blood glucose (FBG), fasting blood insulin (FBI), glycated hemoglobin (HbA1c) and homeostatic model assessment for insulin resistance (HOMA-IR).
2. Studies that reported adjusted relative risk estimates (RR) or hazard ratios (HR) and 95% confidence intervals (for cohort studies); and means, standard deviations (SDs) or standards errors (SEs) or 95% confidence intervals at baseline and/or endpoint for the outcomes investigated (for RCTs).
3. For publications based on one cohort or the same group of participants, only the publication with the longest follow-up years or latest data was included.

## Exclusion criteria

The exclusion criteria were the following:

1. Studies that only examined the effect of individual grain components, including bran, germ, or fiber in endosperm，which have nutritional structural differences compared to whole grains as defined by the HEALTHGRAIN Consortium.
2. Cross-sectional studies or quasi-experimental studies.
3. RCTs with crossover design but reported the outcomes in separate phases.
4. Studies conducted in children or adolescents or in participants with diseases other than metabolic disease (type 2 diabetes, pre-diabetes, metabolic syndrome (MetS), or at risk of metabolic syndrome).
5. Duration of study was less than 2 weeks.

The title and abstract of articles were screened and then full texts of potentially eligible articles were assessed independently by 2 review authors (JZ and TY). The reference lists of the relevant articles were screened to avoid missing any relevant publication.

## Data extraction

Data from the eligible studies were extracted and summarized into separate tables based on study design (cohort studies and RCTs). For both study designs, we collected data on the first author’s name, publication year, country, sample size, sex and age of participants. For prospective cohort studies, additional data were extracted, including: enrollment date, follow-up years, method of dietary intake assessment, method of reporting whole grain intake, whole grain intake levels, total number of cases, risk ratio of type 2 diabetes in the models with the most adjusted confounders. For RCTs, the following additional information were extracted: study design, intervention duration, health status, number of participants in the treatment and control groups, method of whole grain intervention, whole grain intervention dose, baseline and outcome data in FBG, FBI, HbA1c and HOMA-IR, baseline triglycerides (TG) and baseline body mass index (BMI). Data from eligible studies were extracted independently by 2 review authors (TY and WL).

## Risk-of-bias assessment

The quality of the cohort studies was assessed using the validated Newcastle-Ottawa Scale (NOS) tool ^[1]^, which awarded a maximum of nine points to each cohort study: four for quality of selection, two for comparability, and three for the quality of outcome and adequacy of follow-up. We considered studies with scores ≥7 as low risk of bias, scores between 3 and 6 are almost acceptable, but studies with scores ≤3 scores indicating a high risk of bias. The risk of bias for each study was presented in **Supplemental Table 2**. For RCTs, we used the Cochrane Risk of Bias Assessment tool ^[2]^. The domains of assessment were the following: random sequence generation, allocation concealment, reporting bias, performance bias, detection bias, attrition bias, and other sources of bias. Within each domain, a “high risk” score would be given if it contained methodological flaws that might have affected the results and a “low risk” score would be given if the flaw was deemed inconsequential, and “unclear risk” scores would be given if the information was insufficient to determine the impact. Although most RCTs did not implement blind intervention on subjects, it must be recognized this is not usually possible in dietary studies. Apart from allocation concealment and blinding of participants and personnel, if an RCT has a high risk of bias in any other domain, it was considered to have an overall high risk of bias. If there were more than two domains rated as uncertain, the overall risk was classified as uncertain; otherwise, it was categorized as low risk. Evaluation of the risk of bias was conducted by 2 review authors (WL and TY), and disagreements were resolved by consensus-based discussions by all authors. The risk of bias for each study was presented in **Supplemental Table 3.**

## Data synthesis

For the purpose of evaluating the relationship between whole grains and the occurrence of type 2 diabetes in cohort studies, RRs and relevant 95% CIs were used to calculate the overall effect size using a random effect model and hazard ratios were considered approximate measures of RRs. Study-specific regression slopes (linear trends) and variances (95% CIs) were estimated using the method described by Greenland and Longnecker [3], computing the natural logs of the observed RRs across the quantitative exposure categories of the whole grains. The median or mean level of grain intake in each category was assigned to the corresponding RR for each study. If the study reported only the total number of cases and the exposure was defined in categories, we obtained the number of cases in each category by dividing the total number of cases by category numbers, as it was previously described [4]. When the highest or lowest category was open-ended, we assumed the open-ended interval length to be the same as the adjacent interval. When the studies reported data by sex, we pooled the relative risks using a fixed effects model before the meta-analysis.

For RCTs, mean differences for comparing the glycemic biomarkers between the intervention and control groups were used to calculate the overall effect size using a random effect model. When mean differences were not reported, we calculated them by subtracting baseline data from outcome data and the SD for mean differences was imputed using a pooled correlation coefficient according to a published method ^[5]^. The baseline to post-intervention correlation was assumed to be 0.7, which was estimated from the included studies in which all required SDs were reported. Intervention groups were combined to create a single pair-wise comparison to overcome the unit-of-analysis error for studies with

multiple and correlated comparisons ^[6]^.

For both prospective cohort studies and RCTs, *I^2^* statistic was used to assess between-study heterogeneity and a value over 50% indicated a significant level of heterogeneity. One-study-removed sensitivity analyses were obtained to determine whether removing any study could cause significant changes to the results. A further sensitivity analysis was performed to determine the impact of the assumption that correlation between baseline and follow-up SDs was 0.7, in which meta-analysis of FBG was repeated using a correlation of 0.5 and followed by 0.9.

Another sensitivity analysis was performed to determine the impact of inclusion criteria for studies based on the same group of participants, and the meta-analysis of FBG was repeated by replacing studies of longest follow-up years or latest data with shorter and earlier ones. The possibility of a publication bias was examined by the visual inspection of funnel plots and the application of Begg’s test.

For the dose-response analysis, all whole grain intakes were transformed into whole grain ingredients in g/day. There were 4 ways of reporting whole grain intake (whole grain product consumption in g/day or servings/day, or whole grain ingredient consumption in g/day or servings/day). Whole grain ingredients referred specifically to the whole grains contained in whole grain products. To convert servings to grams, it was assumed that one serving of whole grain was equivalent to 30 g. When whole grain ingredient consumption was not reported but whole grain product consumption was reported, it was estimated that whole grain products contained on average 51% of whole grain ingredient,

according to the whole grain food definition ^[7]^. And also, for RCTs reporting both whole grain ingredient and product consumption, the ratio of whole grain ingredients compared to whole grain products was equal to or slightly more than 50% ^[8-11]^. Whole grain ingredients in cooked rice were assumed to weigh 2.25 times as much as uncooked rice ^[12]^. Dose-response relationships between whole grain intakes and outcomes were tested with random effects meta-regression models and restricted cubic splines models ^[13,^ ^14]^. Three fixed knots were used within the total range of the reported intake at 10%, 50% and 90%.

For RCTs, we did further subgroup analysis to detect probable sources of heterogeneity with a random effects model. Subgroup analyses included stratification for lengths of intervention (< 12 week or ≥ 12 week), types of study design (parallel or crossover), whole grain products variety (1-2 types or ≥ 5 types of whole grain products), types of whole grain (wheat, rice, mix or others), health status (generally healthy or unhealthy), baseline BMI, baseline age, baseline triglycerides. Types of whole grain products referred to several major food categories

containing whole grain ingredient, including bread (bread rolls, muffins, biscuits, etc.), cereal (ready-to-eat and hot cereal), grains (pasta, rice, etc.), grain-based desserts (cookies, cakes, pies, chips, etc) and mixed dishes (pizza, salads, etc.). “Generally healthy subgroup” included healthy individuals as well as overweight or obese people, and “unhealthy subgroup” included studies on individuals with pre-diabetes, diabetes, metabolic syndrome, or at risk of metabolic disease (participants with at least one of impaired glucose, lipid or blood pressure). Subgroup

analyses required ≥3 studies in a strata and were not performed when there were insufficient numbers of studies. All analyses were conducted using R 4.0.2 software with “metafor” and “dosresmeta” package. *P* values < 0.05 were considered as statistically significant.

**Supplementary Table 1** PICO tables: whole grains on glycemic control

**Populations**

Adults of any sex without diseases other than metabolic disease (type 2 diabetes, pre-diabetes, metabolic syndrome (MetS), or at risk of metabolic syndrome).

**Subgroup analyses in RCTs where data are available:**

| Health status | “Generally healthy subgroup” included healthy individuals as well as overweight or obese people, and “unhealthy subgroup” included studies on individuals with pre-diabetes, diabetes, metabolic syndrome, or at risk of metabolic disease (participants with at least one of impaired glucose, lipid or blood pressure). |
| --- | --- |
| Baseline age | Age < 50，age > 50 |
| Baseline BMI | BMI < 30 kg/m^2^, BMI ≥ 30 kg/m^2^ |
| Baseline triglycerides | TG ≥ 1.7 mmol/L, TG < 1.7 mmol/L |

**Interventions**

In randomized controlled trials (RCTs), participants were provided with whole grains for a minimum of 3 weeks, or high consumption of whole grains was observed in cohort studies.

**Subgroup analyses or meta regression in RCTs where data are available:**

| Intervention duration | ≥ 12w, < 12w |
| --- | --- |
| Whole grain products variety | 1-2, ≥ 5 |
| Whole grain types | Rice, wheat, others, mix |
| Whole grain doses | Meta-regression and restricted cubic splines models |

**Comparison**

In RCTs, participants were provided with refined grains for a minimum of 3 weeks, or low consumption of whole grains was observed in cohort studies.

**Subgroup analyses in RCTs where data are available:**

| Trail design | Parallel, crossover |
| --- | --- |
| Study quality | Low risk, uncertain, high risk |

**Outcomes:**

| Primary outcomes | Change in fasting blood glucose (FBG) |
| --- | --- |
| Primary outcomes | Change in fasting blood insulin (FBI) |
| Primary outcomes | Change in glycated hemoglobin (HbA1c) |
| Primary outcomes | Change in homeostatic model assessment for insulin resistance (HOMA-IR) |
| Primary outcomes | Risk of type 2 diabetes |

**Supplementary Table 2** Quality assessments of included prospective cohort studies by the validated Newcastle-Ottawa Scale (NOS) tool

| **Author, year** | **Exposed cohort represent**  **ative** | **Nonexposed cohort representative** | **Exposure ascertainm ent** | **Outcome not present at start** | **Controlle d for weight** | **Controll ed for age** | **Outcome assessmen t** | **Follow up duration** | **Follow up adequ**  **acy** | **Total Score^a^** |
| --- | --- | --- | --- | --- | --- | --- | --- | --- | --- | --- |
| Mayer et al., 2000 | Yes | Yes | No | Yes | Yes | Yes | No | No | No | 5 |
| Montonen et al.,  2003 | Yes | Yes | No | Yes | Yes | Yes | Yes | Yes | No | 7 |
| van Dam et al.,  2006 | No | Yes | No | Yes | Yes | Yes | No | Yes | Yes | 6 |
| Ericson et al., 2013 | Yes | Yes | Yes | Yes | Yes | Yes | Yes | Yes | Yes | 9 |
| Packer et al., 2013 | No | Yes | No | Yes | Yes | Yes | Yes | Yes | Yes | 7 |
| Wirstrom et al.,  2013 | No | Yes | Yes | Yes | Yes | Yes | No | Yes | No | 6 |
| Kyro et al., 2018 | Yes | Yes | No | Yes | Yes | Yes | Yes | Yes | Yes | 8 |
| Hu et al., 2020a | No | Yes | Yes | Yes | Yes | Yes | Yes | Yes | No | 7 |
| Hu et al., 2020b | No | Yes | Yes | Yes | Yes | Yes | Yes | Yes | No | 7 |
| Hu et al., 2020c | No | Yes | Yes | Yes | Yes | Yes | Yes | Yes | No | 7 |

^a^Studies with scores ≥7 are recognized as low risk of bias, scores between 3 and 6 are almost acceptable, studies with scores ≤3 indicate a high risk of bias

**Supplementary Table 3** Quality assessments of included RCTs by the Cochrane Risk of Bias Assessment tool

| **Author, year** | **Random sequence generation** | **Allocation concealment** | **Blinding of participants and**  **personnel** | **Blinding of outcome**  **assessment** | **Incomplete outcome data** | **Selective reporting** | **Other bias** | **Overall**  **risk** |
| --- | --- | --- | --- | --- | --- | --- | --- | --- |
| Xue et al., 2021 | L | U | U | L | L | L | L | L |
| Ren et al., 2020 | U | U | H | L | L | L | L | L |
| Mai et al., 2020 | U | U | H | L | L | L | L | L |
| Roager et al., 2019 | L | L | U | L | L | L | L | L |
| Malik et al., 2019 | U | U | H | L | H | L | L | H |
| Kuroda et al., 2019 | U | U | H | L | H | L | L | H |
| Hoevenaars et al., 2019 | L | L | U | L | L | L | L | L |
| Kikuchi et al., 2018 | U | U | L | L | L | L | L | L |
| Karl et al., 2017 | U | L | H | L | U | L | L | L |
| Nakayama et al., 2017 | U | U | H | L | L | U | U | U |
| Kristensen et al., 2017 | U | U | H | L | L | L | H | H |
| Kondo et al., 2017 | L | L | H | L | L | L | L | L |
| Cooper et al., 2017 | U | U | H | L | U | U | L | U |
| Vetrani et al., 2016 | L | L | H | L | L | L | U | L |
| Kirwan et al., 2016 | U | U | L | L | L | L | L | L |
| Geng et al., 2016 | U | U | H | L | H | L | L | H |
| Connolly et al., 2016 | L | L | U | L | L | L | L | L |
| Ampatzoglou et al.,  2016 | L | U | H | L | L | L | L | L |
| Vitaglione et al., 2015 | L | L | H | L | L | L | L | L |
| Jackson et al., 2014 | L | H | H | L | L | L | L | H |

| Bui et al., 2014 | U | U | H | L | L | L | L | L |
| --- | --- | --- | --- | --- | --- | --- | --- | --- |
| Wang et al., 2012 | U | U | H | L | U | L | L | U |
| Mackay et al., 2012 | L | U | H | L | L | U | L | L |
| Kristensen et al., 2012 | U | U | H | L | L | L | L | L |
| Zhang et al., 2011 | U | U | H | L | L | L | L | L |
| Tighe et al., 2010 | U | U | H | L | L | L | L | L |
| Giacco et al., 2010 | U | U | H | L | L | L | L | L |
| Brownlee et al., 2010 | L | L | H | L | L | L | L | L |
| Kim et al., 2008 | U | U | U | L | L | L | L | L |
| Katcher et al., 2008 | U | U | H | L | L | L | U | U |
| Andersson et al., 2007 | U | U | H | L | L | L | L | L |
| McIntosh et al., 2003 | U | U | H | L | H | L | L | H |
| Li et al., 2003 | H | U | H | L | L | U | L | H |
| Pins et al., 2002 | U | U | H | L | L | L | L | L |
| Pereira et al., 2002 | U | U | H | L | L | U | L | U |
| Hawrysh et al., 1998 | U | U | H | L | L | U | L | U |

^a^H, high; L, low; U, uncertain

^b^ Although most RCTs did not implement blind intervention on subjects, it must be recognized this is not usually possible in dietary studies. Apart from allocation concealment and blinding of participants and personnel, if an RCT has a high risk of bias in any other domain, it was considered to have an overall high risk of bias. If there were more than two domains rated as uncertain, the overall risk was classified as uncertain; otherwise, it was categorized as low risk.


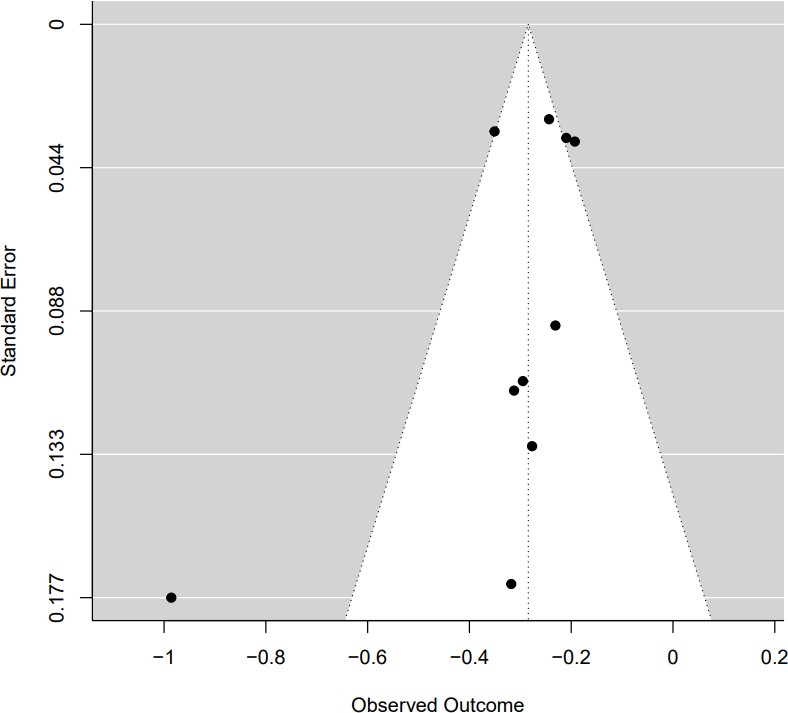


**Supplementary Fig. 1** Funnel plot for the effects of whole grains on risk of T2D in adults


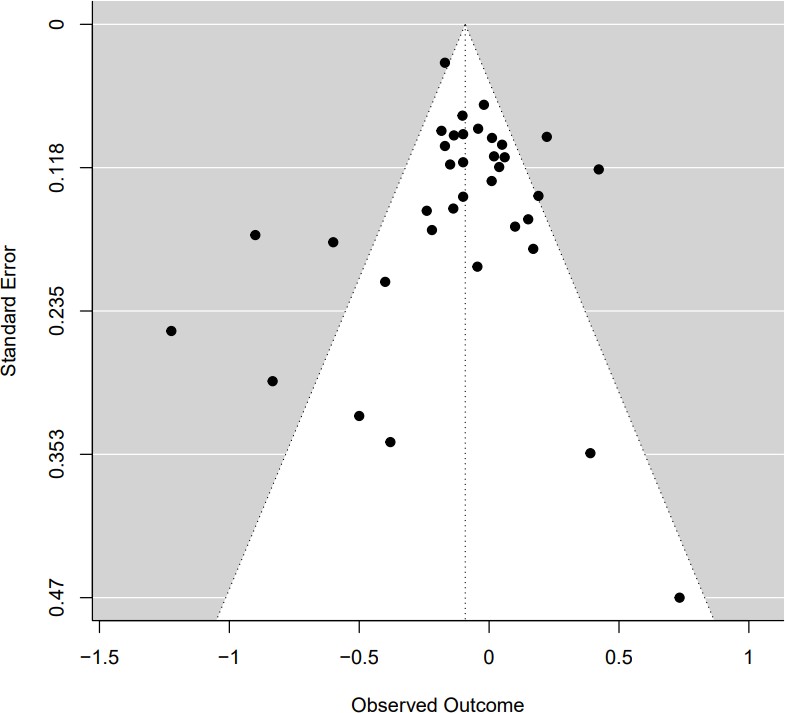


**Supplementary Fig. 2** Funnel plot for the effects of whole grains on FBG in adults


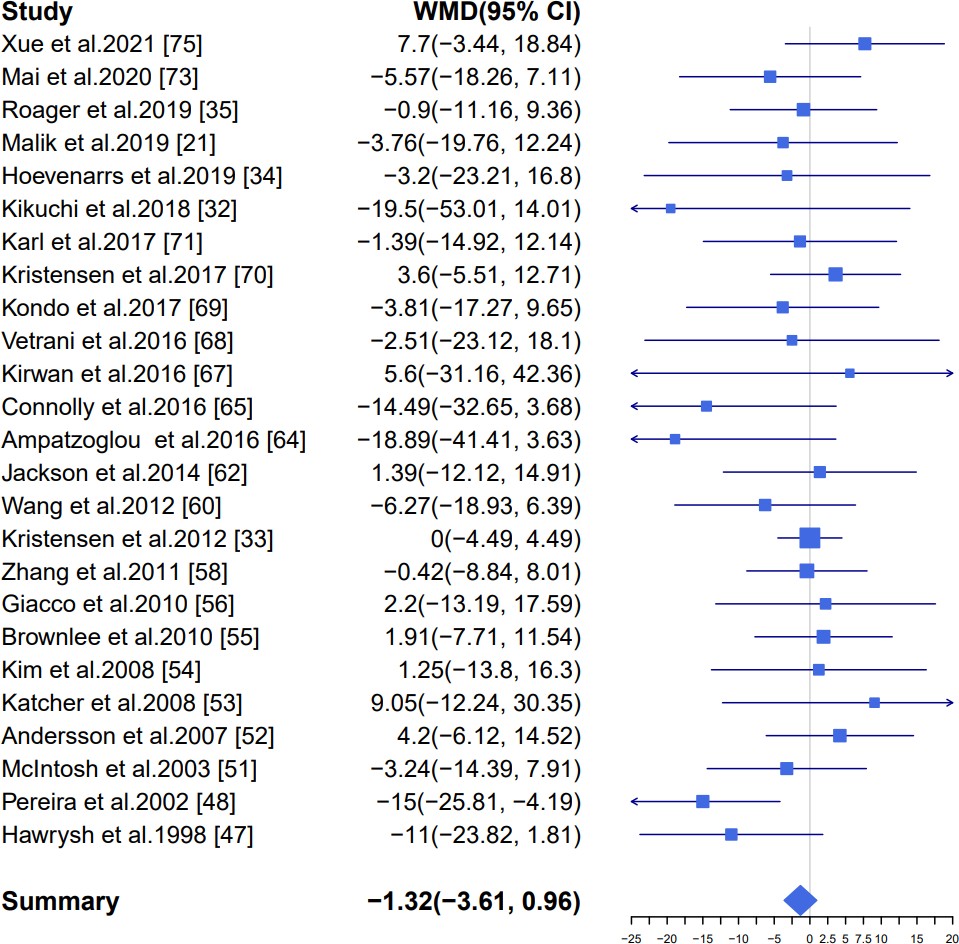


**Supplementary Fig. 3** Forest plot for the effects of whole grains on fasting blood insulin in adults, expressed as mean differences between intervention and control groups. The area of each square is proportional to the inverse of the variance of the weighted mean difference. Horizontal lines represent 95% CIs


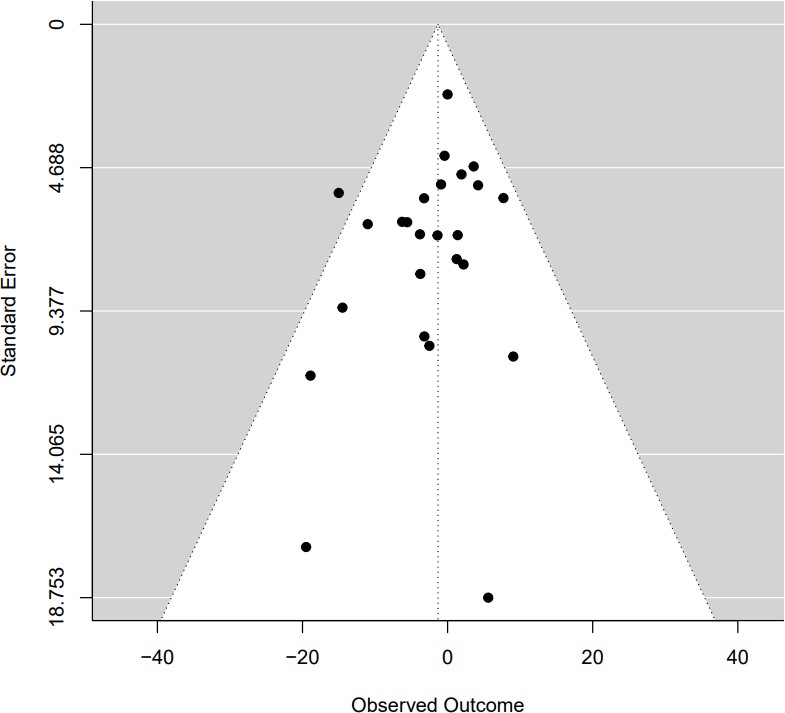


**Supplementary Fig. 4** Funnel plot for the effects of whole grains on fasting blood insulin in adults


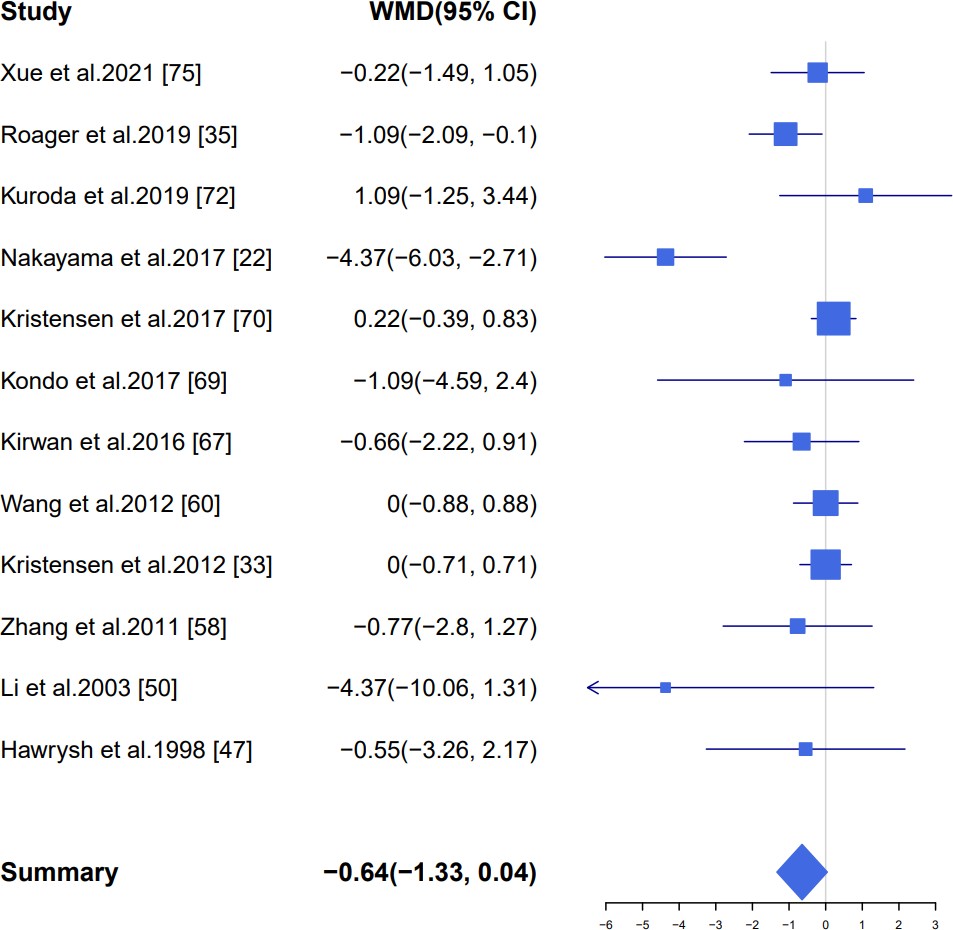


**Supplementary Fig. 5** Forest plot for the effects of whole grains on HbA1c in adults, expressed as mean differences between intervention and control groups. The area of each square is proportional to the inverse of the variance of the weighted mean difference. Horizontal lines represent 95% CIs


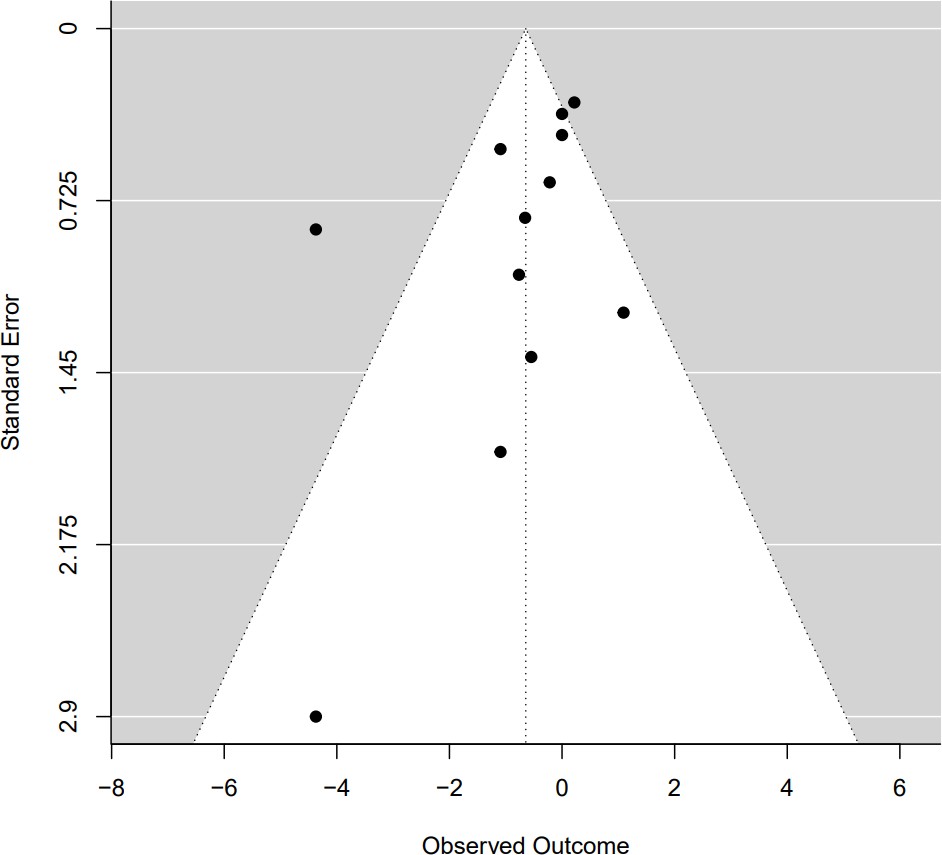


**Supplementary Fig. 6** Funnel plot for the effects of whole grains on HbA1c in adults


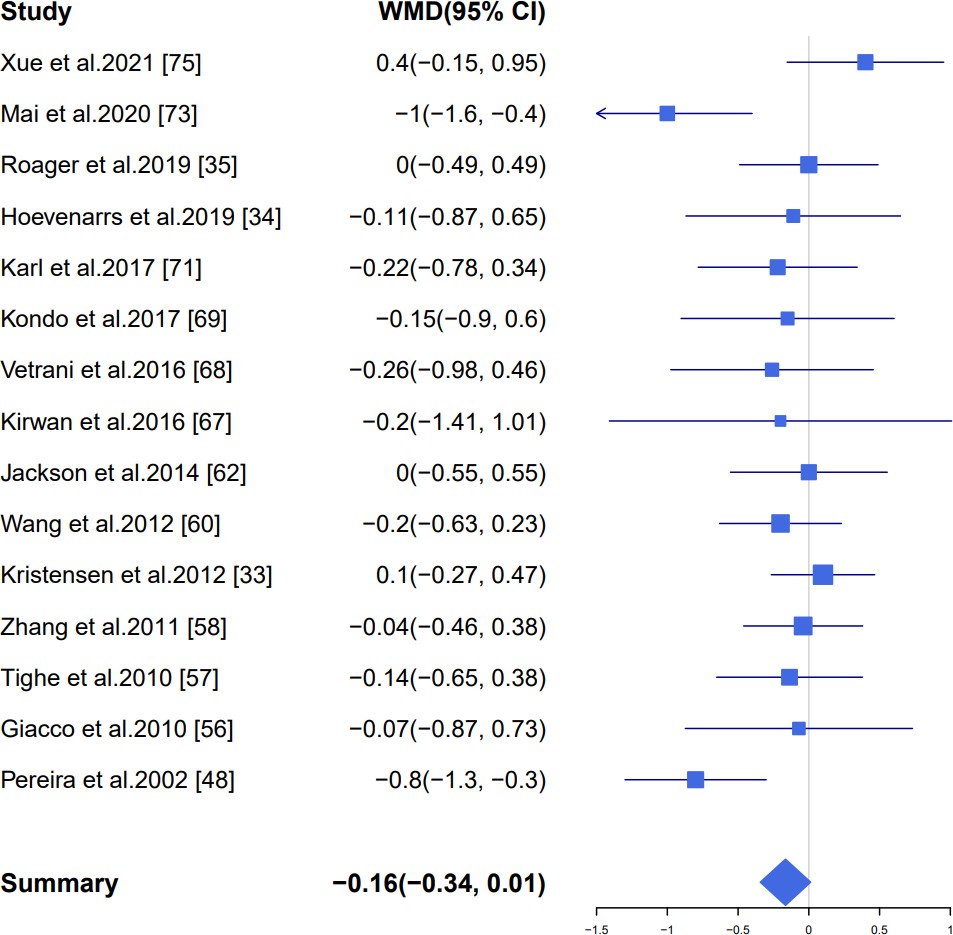


**Supplementary Fig. 7** Forest plot for the effects of whole grains on HOMA-IR in adults, expressed as mean differences between intervention and control groups. The area of each square is proportional to the inverse of the variance of the weighted mean difference. Horizontal lines represent 95% CIs


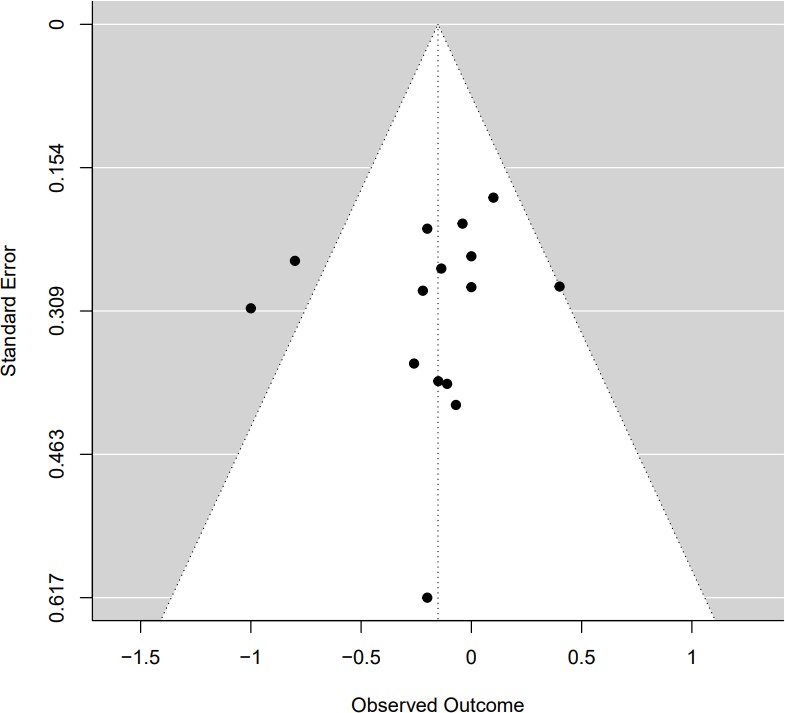


**Supplementary Fig. 8** Funnel plot for the effects of whole grains on HOMA-IR in adults

1. WELLS G.A., SHEA B., O’CONNELL D., et al., *The Newcastle-Ottawa Scale (NOS) for assessing the quality of nonrandomised studies in meta-analyses*. 2000, Oxford.
2. HIGGINS J.P.T., ALTMAN D.G., GøTZSCHE P.C., et al., The Cochrane Collaboration’s tool for assessing risk of bias in randomised trials[J]*.* BMJ. 2011. **343**: d5928. [3]GREENLAND S.,LONGNECKER M.P., Methods for trend estimation from summarized dose-response data, with applications to meta-analysis[J]*.* Am. J. Epidemiol. 1992.

**135**(11): 1301-9.

1. MORZE J., SCHWEDHELM C., BENCIC A., et al., Chocolate and risk of chronic disease: a systematic review and dose-response meta-analysis[J]*.* Eur. J. Nutr. 2020. **59**(1): 389-397.
2. CUMPSTON M., LI T., PAGE M.J., et al., Updated guidance for trusted systematic reviews: a new edition of the Cochrane Handbook for Systematic Reviews of Interventions[J]*.* Cochrane Database Syst. Rev. 2019. **10**: Ed000142.
3. HIGGINS J.P., THOMAS J., CHANDLER J., et al., *Cochrane handbook for systematic reviews of interventions*. 2019: John Wiley & Sons.
4. VAN DER KAMP J.W., POUTANEN K., SEAL C.J., et al., The HEALTHGRAIN definition of 'whole grain'[J]*.* Food Nutr. Res. 2014. **58**.
5. KIKUCHI Y., NOZAKI S., MAKITA M., et al., Effects of whole grain wheat bread on visceral fat obesity in Japanese subjects: A randomized double-blind study[J]*.* Plant Foods Hum. Nutr. 2018. **73**(3): 161-165.
6. KRISTENSEN M., TOUBRO S., JENSEN M.G., et al., Whole grain compared with refined wheat decreases the percentage of body fat following a 12-week, energy-restricted dietary intervention in postmenopausal women[J]*.* The Journal of nutrition. 2012. **142**(4): 710-716.
7. HOEVENAARS F.P.M., ESSER D., SCHUTTE S., et al., Whole Grain Wheat Consumption Affects Postprandial Inflammatory Response in a Randomized Controlled Trial in Overweight and Obese Adults with Mild Hypercholesterolemia in the Graandioos Study[J]*.* J. Nutr. 2019. **149**(12): 2133-2144.
8. ROAGER H.M., VOGT J.K., KRISTENSEN M., et al., Whole grain-rich diet reduces body weight and systemic low-grade inflammation without inducing major changes of the gut microbiome: a randomised cross-over trial[J]*.* Gut. 2019. **68**(1): 83-93.
9. GOLZARAND M., TOOLABI K., ESKANDARI DELFAN S., et al., The effect of brown rice compared to white rice on adiposity indices, lipid profile, and glycemic markers: a systematic review and meta-analysis of randomized controlled trials[J]*.* Crit. Rev. Food Sci. Nutr. 2021: 7395-7412.
10. ORSINI N., BELLOCCO R.,GREENLAND S., Generalized Least Squares for Trend Estimation of Summarized Dose–response Data[J]*.* The Stata Journal. 2006. **6**(1): 40-57.
11. SHIM S.R.,LEE J., Dose-response meta-analysis: application and practice using the R software[J]*.* Epidemiology and health. 2019. **41**: e2019006-e2019006.
